# p53 Orchestrates the Immunogenic-Tolerogenic Pyroptosis Switch in Non-Small Cell Lung Cancer: A Systems Biology Approach

**DOI:** 10.34133/csbj.0172

**Published:** 2026-07-21

**Authors:** Shantanu Gupta, Daner A. Silveira, Rodrigo Juliani Siqueira Dalmolin, José Carlos M. Mombach, Ronaldo F. Hashimoto

**Affiliations:** ^1^ Bioinformatics Multidisciplinary Environment-BioME—Digital Metropole Institute, Federal University of Rio Grande do Norte, Natal 59076550, RN, Brazil.; ^2^ Children’s Cancer Institute, Porto Alegre, Rio Grande do Sul, Brazil.; ^3^ Departamento de Física, Universidade Federal de Santa Maria, Santa Maria 97105-900, RS, Brazil.; ^4^ Departamento de Ciência da Computação, Instituto de Matemática, Estatística e Ciência da Computação, Universidade de São Paulo, Rua do Matão 1010, 05508-090 São Paulo, SP, Brazil.

## Abstract

Resistance to immunogenic cell death drives therapy failure in non-small cell lung cancer (NSCLC). While the tumor suppressor p53 can activate both canonical (NLRP3–caspase-1–GSDMD) and secondary (caspase-9/3–GSDME) pyroptosis, 2 lytic programs with divergent immunogenicity, the systems-level logic coordinating these parallel execution pathways remains unknown, limiting therapeutic exploitation. Here, we deploy, to our knowledge, the first dynamic Boolean network model of p53-regulated pyroptosis in NSCLC to resolve this decision layer. The model suggests that the terminal caspase–gasdermin axis may be structured as a bistable immunogenic switch, governed by interlocked feedback loops: a double-negative motif (caspase-9–caspase-3–GSDMD) that enables mutual exclusivity and a reinforcing loop (GSDME–caspase-9–caspase-3) that commits to secondary pyroptosis. Within the model, this topology positions GSDME not as a passive executioner but as a critical fate-determining node; its loss, frequent in NSCLC, does not abort death signaling but is predicted to re-route p53-engaged caspase-3 activity toward apoptosis, providing a potential explanation for how tumors may evade immunogenic lysis while retaining apoptotic competence. Model predictions are validated against NSCLC cell-line phenotypes and patient transcriptomics, revealing coordinated repression of pyroptosis-execution genes and identifying *CASP9/GSDME* as adverse prognostic markers. Collectively, our results suggest that p53 functions as a central coordinator of a terminal fate-switch network rather than a simple linear activator and provide a systems-level framework for investigating how execution-layer regulatory circuits influence cell-death outcomes in NSCLC.

## Introduction

Non-small cell lung cancer (NSCLC) remains one of the deadliest malignancies worldwide, with therapy resistance emerging as the dominant barrier to durable responses [1]. While evasion of apoptosis, a generally tolerogenic form of programmed cell death, through BCL2 apoptosis regulator (BCL-2) family deregulation or caspase inhibition is a well-recognized hallmark of NSCLC [2], growing evidence indicates that tumors also actively suppress more immunogenic forms of regulated cell death (RCD) to establish an immunosuppressive microenvironment [3]. Among these, pyroptosis, a lytic, proinflammatory modality driven by gasdermin pore formation and massive release of interleukin-1β (IL-1β) and interleukin-18 (IL-18), has recently emerged as a potent trigger of anti-tumor immunity when successfully executed [4]. In contrast to apoptosis, which is generally tolerogenic, pyroptosis transforms dying cancer cells into potent vaccines capable of recruiting and activating dendritic cells and cytotoxic T lymphocytes [5]. However, NSCLC cells frequently disable pyroptotic machinery, contributing to both intrinsic and acquired resistance to chemo-, radio-, and immunotherapy [6].

The tumor suppressor p53 (p53) is a central integrator of cellular stress responses in NSCLC and a key regulator of multiple cell-death pathways beyond classical apoptosis [7]. Given that approximately half of NSCLC tumors retain wild-type (WT) p53 or harbor mutations that partially preserve transcriptional activity [7] this study focuses on p53-driven pyroptosis regulation in the context of functional p53 signaling. In its apoptotic role, p53 transcriptionally activates pro-apoptotic genes such as p53 up-regulated modulator of apoptosis (PUMA), NOXA, and BCL2-associated X protein (BAX), leading to mitochondrial outer membrane permeabilization, caspase-9/3 activation, and ultimately tolerogenic apoptosis, a controlled, non-inflammatory cell death that avoids immune activation. Recent studies show that WT p53 can also activate both canonical and secondary pyroptosis in NSCLC through phosphorylation-dependent functional states [8,9]. While low p53 activity preferentially engages the p53–p21 axis to enforce cell-cycle arrest and senescence [10,11], high p53 activity licenses a death-competent p53 program that induces both inflammasome priming via NLR family pyrin domain containing 3 (NLRP3) [8,12] and mitochondrial apoptosis signaling through PUMA and BAX [10]. PUMA-mediated inhibition of BCL2 enables BAX activation and downstream caspase-9/3 signaling, coupling mitochondrial stress to gasdermin E (GSDME) cleavage and secondary pyroptosis, while parallel activation of the NLRP3–caspase-1–gasdermin D (GSDMD) axis drives canonical pyroptotic execution [8,9]. Importantly, these 2 pyroptotic pathways exhibit markedly different immunogenicity profiles: Canonical pyroptosis is strongly pro-inflammatory, whereas secondary pyroptosis and apoptosis are progressively tolerogenic [13]. Despite these advances, the systems-level logic determining how p53 simultaneously engages parallel executioner modules, and how tumors rewire this decision toward less immunogenic outcomes, remains unknown.

This gap is critical because canonical and secondary pyroptosis share upstream p53 activation yet diverge at the execution layer, where feedback regulation, signal integration, and competition between caspase–gasdermin modules are likely to determine cell-fate commitment. Capturing such nonlinear decision-making requires a systems biology framework that moves beyond sequential pathway descriptions toward an explicit representation of regulatory logic and feedback structure. Boolean network modeling offers a well-established computational–experimental integrative approach for this purpose, enabling qualitative analysis of complex regulatory systems in which stable attractors correspond to distinct cellular phenotypes. Seminal studies established frameworks for apoptosis [14], while subsequent work introduced Boolean models of ferroptosis [15] and autophagy [16]. To date, however, no Boolean model has been developed for pyroptosis pathway crosstalk. Here, we present the first dynamic Boolean network model of p53-regulated pyroptosis in NSCLC, integrating both canonical and secondary pathways with apoptotic crosstalk. Our analysis reveals a bistable immunogenic switch governed by interlocked feedback loops and uncovers qualitative rewiring of this topology in NSCLC tumors with prognostic implications.

Although this study focuses on NSCLC, the core regulatory logic identified here is not inherently tissue-specific. The caspase–gasdermin modules likely operate in other epithelial cancers where p53, inflammasome, and mitochondrial death pathways converge—as supported by evidence from breast [17], colorectal [18], and gastric [19] cancers. NSCLC thus serves as a well-characterized model system to uncover principles applicable across multiple tumor types.

## Materials and Methods

### Mapping cancer gene networks with public data and tools

We constructed a mechanistic gene regulatory network describing p53-driven pyroptosis under DNA damage conditions in NSCLC. Experimentally validated molecular interactions were identified through systematic manual curation of the literature using PubMed and cross-referenced with curated interaction resources, including BIOGRID v3.5 (https://thebiogrid.org/) [20]. Only interactions supported by direct experimental evidence were retained. Whenever possible, interactions were sourced from primary literature with direct biochemical or genetic evidence. In cases where primary sources were difficult to access, review articles were consulted to trace the original studies supporting the interaction. The curated network was implemented as a Boolean model using GINsim 3.0.0b (https://ginsim.github.io/install/) [21]. The model file is provided in the “Data Availability” section.

### Boolean modeling, asynchronous updating, and perturbation analysis

The curated regulatory network was formalized as a Boolean dynamical system, in which nodes represent molecular species, protein complexes, or cellular processes, and edges denote activating or inhibitory regulatory interactions. Each node assumes a binary state, inactive (0) or active (1), and its state is determined by a logical rule (AND, OR, NOT) derived from experimentally supported regulatory relationships [22]. Boolean update rules were defined using standard logical operators and are provided in Table S1.

All nodes represent binary activation states (1 = active, 0 = inactive), where “active” is defined as the functional form sufficient to exert downstream effects, whether through expression (e.g., PUMA) or posttranslational cleavage (e.g., caspases and gasdermins).

Simulation of the Boolean network generates a state transition graph (STG), where nodes correspond to global network states and edges represent possible state transitions. Stable-state attractors, defined as terminal states with no outgoing transitions except self-loops, were interpreted as distinct cellular phenotypes, whereas cyclic attractors correspond to oscillatory or transient behaviors [22]. Feedback loops (circuits) within the regulatory graph are key determinants of network dynamics: Positive feedback loops are responsible for multi-stability, while negative feedback loops contribute to oscillations [23].

Network dynamics were explored using asynchronous updating, in which only one node is updated at each simulation step, reflecting the intrinsic stochasticity and lack of global synchronization in cellular regulatory systems. To estimate phenotype probabilities, Monte Carlo simulations were performed by generating large ensembles of random asynchronous trajectories from unbiased initial conditions [22]. The probability of each attractor was calculated as the fraction of trajectories converging to that state.

Gain- and loss-of-function perturbations were implemented directly within the Boolean framework by fixing selected nodes in the active (1) or inactive (0) state, respectively, independently of their regulatory inputs [22]. For more details about the Boolean modeling, see our previous studies [24–27].

### Expression analysis of execution-layer genes in NSCLC patient cohorts

To evaluate the clinical relevance of the modeled caspase–gasdermin axis, transcriptomic data from NSCLC patient cohorts was analyzed using the GEPIA3 platform [28], which integrates the Cancer Genome Atlas (TCGA) and Genotype-Tissue Expression (GTEx) RNA-sequencing datasets. A composite execution-layer signature comprising *GSDMD*, *GSDME*, *NLRP3*, *CASP3*, and *CASP9* was examined in lung adenocarcinoma (LUAD) and lung squamous cell carcinoma (LUSC). Expression values were extracted as log₂(TPM + 1), and differential expression between tumor and adjacent normal tissues was assessed using GEPIA3’s limma-based pipeline, which applies moderated *t* tests with Benjamini–Hochberg false discovery rate correction. These genes were selected to represent a minimal execution-layer decision module linking inflammasome priming, mitochondrial caspase signaling, and gasdermin-mediated membrane rupture while excluding upstream sensors and obligate intermediates to maintain focus. Note that gene symbols (e.g., *CASP3* and *CASP9*) are used for transcript-level analyses, whereas protein names (e.g., caspase-3 and caspase-9) denote functional activity in the Boolean network.

### Survival analysis

The prognostic relevance of execution-layer components was evaluated using multivariable Cox proportional hazards regression implemented in the GEPIA3 survival module [28]. The model included the 5 execution-layer genes as continuous predictors. Hazard ratios (HRs) with 95% confidence intervals were calculated to assess the independent prognostic impact of each gene on overall survival. Statistical significance was determined using standard Cox regression procedures.

### TP53-stratified survival analysis

To assess whether the prognostic impact of execution-layer genes depends on TP53 status, gene expression data (RNA-seq V2 RSEM) and somatic mutation calls for LUAD and LUSC cohorts were retrieved from cBioPortal (TCGA PanCancer Atlas 2018) [29]. After merging expression, mutation, and clinical data, a total of 974 patients were included (LUAD, *n* = 497; LUSC, *n* = 477). Patients were classified as TP53-mutant (*n* = 636) or TP53-WT (*n* = 338). Multivariable Cox proportional hazards models were fitted separately for each TP53 subgroup for CASP3, CASP9, GSDMD, GSDME, and NLRP3. Gene expression values were standardized (*z* score) within each subgroup. Histological subtype (LUAD versus LUSC) was included as a covariate. Analyses were performed using R version 4.4.1.

## Results

### The model and WT attractor analysis

The network integrates inflammasome signaling, mitochondrial caspase activation, and gasdermin-mediated membrane permeabilization. Genotoxic stress was abstracted as a single external input (“Drug”), representing DNA-damaging therapies such as ionizing radiation [30], chemotherapy [31], or targeted agents [32]. The drug activates ataxia-telangiectasia mutated (ATM) [33] and adenosine monophosphate-activated protein kinase (AMPK) [34], while WT p53-induced phosphatase 1 (Wip1) inactivates ATM [35] and E2F transcription factor 1 (E2F1) promotes ATM expression [36]. This signaling leads to suppression of mouse double minute 2 Homolog (Mdm2)/Wip1 and stabilization of functionally distinct p53 states [37], with ATM directly phosphorylating and inhibiting Mdm2 [38].

To capture p53 functional plasticity, the model adopts the 2-phase p53 dynamics framework proposed by Zhang et al. [39], explicitly distinguishing low-activity p53 (p53^A^, arrest-associated) from high-activity p53 (p53^K^, death-associated), with p53-inducible nuclear protein 1 (p53INP1) regulating their balance [39]. These activity states are determined by stress duration and regulatory inputs, consistent with the observation that sustained p53 activation favors death outcomes [39]. p53^A^ is stabilized when Mdm2 [40] and Wip1 [41] are inactive, while p53^K^ is phosphorylated and stabilized by ATM [42] and degraded by Mdm2 [40].

p53^A^ induces cyclin-dependent kinase inhibitor 1A (p21), enforcing CdkCyclin– retinoblastoma protein 1 (RB1)–E2F1 inhibition and senescence [43,44]. p21 inhibits caspase-3 [45] and is suppressed by AKT [46]. In parallel, p53^K^, biased by p53INP1 under sustained damage, activates both inflammasome priming (NLRP3) and mitochondrial stress (PUMA) [10,12], with BCL2 inhibiting NLRP3 [47]. NLRP3 signaling engages caspase-1-dependent GSDMD cleavage to execute canonical pyroptosis [12,48], whereas PUMA-mediated mitochondrial permeabilization activates caspase-9 and caspase-3 [10], enabling GSDME-dependent secondary pyroptosis [49,50]. Caspase-3 cleaves GSDME [51] and inhibits GSDMD [52]; GSDMD suppresses caspase-9 [53], while GSDME amplifies caspase-9 via cytochrome c release [17,54]; caspase-9 activates caspase-3 [55]. GSDMD is suppressed by AMPK [56]; GSDME cleavage is promoted by AMPK [57].

BCL-2 inhibits BAX [58,59] and suppresses NLRP3, thereby limiting pyroptotic execution. BCL2 is inhibited by PUMA and stabilized by AKT; cyclin-dependent kinase–cyclin is activated by Cdc25 and inhibited by p21 and ATM–Cdc25 signaling; RB1 phosphorylation frees E2F1 for proliferation [60–65].

To enforce mutual exclusivity between pyroptotic and apoptotic execution, the interaction “GSDME inhibits Apoptosis” was included, reflecting competitive routing of caspase-3 activity [18,51,66]. The mechanistic basis for this representation is explained in Table S1.

**Fig. 1.**
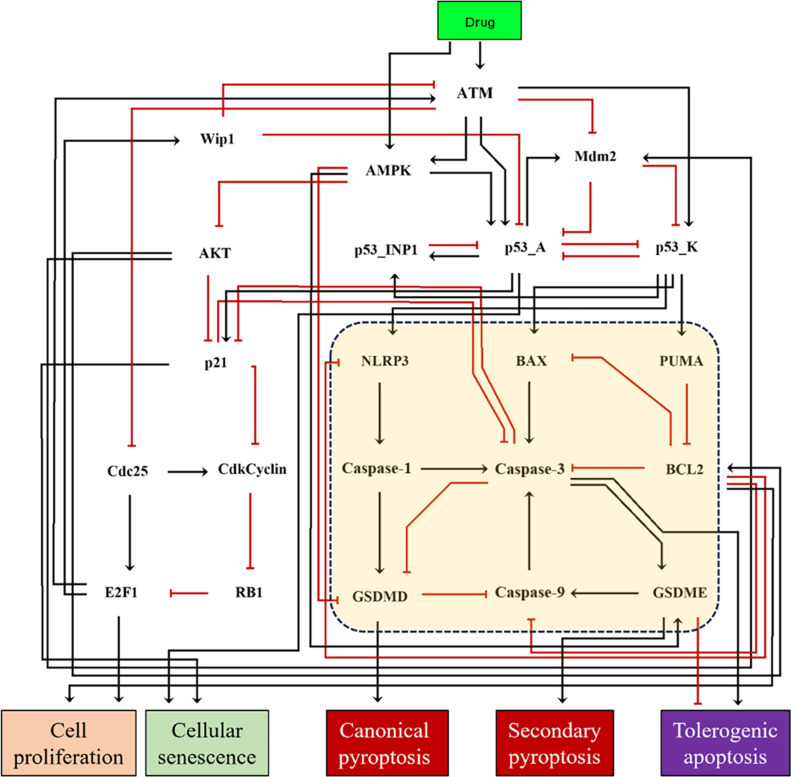
Dynamic Boolean model of p53-mediated regulation of pyroptosis–apoptosis in NSCLC. The regulatory network depicts experimentally validated molecular interactions governing DNA damage-induced outcomes downstream of p53. Black arrows indicate activation; red hammerhead lines denote inhibition. The exogenous input “Drug” (green node) represents genotoxic therapies (e.g., ionizing radiation and chemotherapy). Phenotypic outputs are color-coded at the bottom: proliferation (peach), cellular senescence (light green), canonical pyroptosis (dark red), secondary pyroptosis (dark red), and tolerogenic apoptosis (purple). The core cell death machinery, integrating both inflammatory pyroptotic pathways (canonical and secondary) and non-inflammatory tolerogenic apoptosis, is highlighted in a light beige box.

These mechanistic insights were encoded into a Boolean model to elucidate how p53 orchestrates the pyroptosis switch. The final regulatory graph comprises 28 nodes and 58 direct interactions (Fig. 1).

The “Drug” input is modeled as a binary variable with “ON” and “OFF” states, and proteins are represented as rectangular nodes. Key model outputs, proliferation, senescence, canonical pyroptosis, secondary pyroptosis, and tolerogenic apoptosis are explicitly labeled. Overall, the network features 58 direct interactions among its signaling components, capturing the complexity of p53-mediated cell fate decisions.

**Fig. 2.**
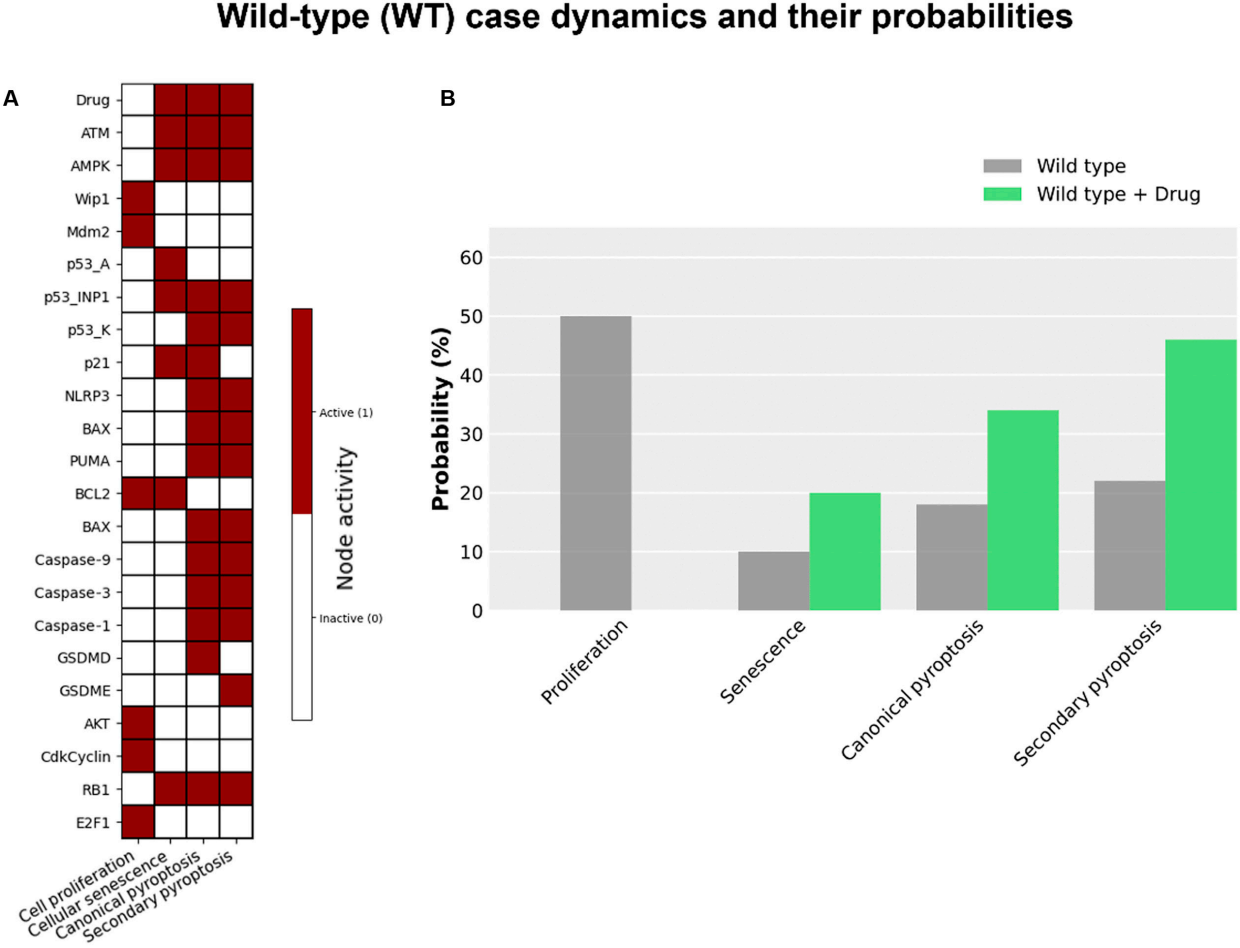
Wild-type attractor dynamics in NSCLC. (A) Each column represents a stable attractor, and each row represents a molecular node. Maroon indicates active state (1); white indicates inactive state (0). Phenotypes are labeled at the bottom: proliferation, senescence, canonical pyroptosis, and secondary pyroptosis. (B) Monte Carlo-derived steady-state phenotypic probabilities (100,000 simulations). Gray bars represent outcome distributions under neutral input conditions (Drug unconstrained), while green bars show distributions when Drug is fixed ON, corresponding to sustained DNA damage.

Simulation of the WT network identified 4 robust attractors (Fig. 2A). In the absence of genotoxic stress (Drug OFF), proliferation dominates and is maintained through coordinated MDM2–Wip1–BCL2–AKT signaling. Upon Drug activation, 3 distinct damage-induced fates emerge: senescence (p53^A^ → p21/BCL2), canonical pyroptosis (NLRP3 → caspase-1 → GSDMD), and secondary pyroptosis (caspase-9 → caspase-3 → GSDME). Tolerogenic apoptosis, though represented in the model, did not emerge as a WT attractor; conditions facilitating apoptotic competence are explored in subsequent sections.

Monte Carlo simulations revealed context-dependent fate probabilities (Fig. 2B). Under neutral input conditions, the system distributes across proliferation (50%), senescence (10%), canonical pyroptosis (22%), and secondary pyroptosis (18%). The nonzero probability of death phenotypes under neutral conditions reflects basal endogenous stress signaling in cancer cells (e.g., oncogenic reactive oxygen species and replication stress), consistent with observed spontaneous low-level RCD in untreated NSCLC populations [49] and general endogenous stress in tumor microenvironments.

When Drug is fixed ON, the system shifts entirely toward damage-responsive fates, with secondary pyroptosis (46%) and canonical pyroptosis (34%) dominating over senescence (20%). These results indicate that sustained DNA damage redistributes cell fate toward pyroptotic outcomes without enforcing a single dominant execution mode.

### Model validation and calibration against experimental phenotypes in NSCLC cell lines

**Fig. 3.**
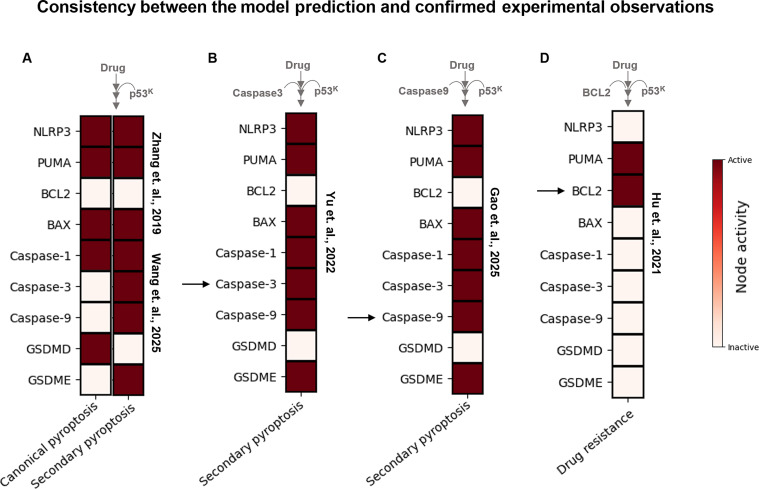
Model validation via gain-of-function (GoF) perturbation analysis. Model predictions under various mutant conditions are compared with established experimental phenotypes. Each column represents a stable state; each row shows the activity level of a molecular node. (A) Overexpression of p53^K^ induces canonical/secondary pyroptosis. (B) Caspase-3 overexpression leads to secondary pyroptosis. (C) Caspase-9 overexpression triggers secondary pyroptosis. (D) BCL2 overexpression blocks pyroptosis and promotes survival/drug resistance. Experimental support for each prediction is detailed in the Model validation and calibration against experimental phenotypes in NSCLC cell lines section.

Model fidelity was rigorously evaluated through systematic in silico gain- and loss-of-function perturbations performed under p53^K^-dominant conditions, thereby isolating pyroptosis-permissive dynamics from senescence. Predicted outcomes were benchmarked against established experimental phenotypes in NSCLC models (see Fig. 3 and Table S2 for details).

Notably, while the network was constructed from a curated set of experimentally validated interactions, model validation employed independent studies not utilized during network construction, ensuring unbiased assessment of predictive accuracy (Table S3 provides a detailed mapping of each validation study alongside the primary interaction sources for complete transparency).

Constitutive activation of the p53^K^ state yielded stable coexistence of canonical pyroptosis (via the NLRP3–caspase-1–GSDMD axis) and secondary pyroptosis (via the caspase-9/3–GSDME axis), recapitulating the experimental observations by Zhang et al. [8] and Wang et al. [9] across multiple cancer models, including NSCLC (see Fig. 3A). Enforced activation of caspase-3 collapsed this executional heterogeneity and locked the system into secondary pyroptosis through direct GSDME cleavage, consistent with reports of caspase-3-dependent pyroptotic lysis by Yu et al. [49] in a panel of NSCLC cell lines, including NCI-H226, SK-MES-1, A549, NCI-HCC827, NCI-H1975, and PC9 (see Fig. 3B). Similarly, constitutive caspase-9 activation drove secondary pyroptosis via downstream caspase-3–GSDME signaling, in agreement with the mitochondrial caspase engagement observed by Gao et al. [67] in A549 and H1299 cells (see Fig. 3C). BCL-2 overexpression suppressed both pyroptotic branches by restraining mitochondrial caspase activation, consistent with the anti-pyroptotic role of BCL-2 family proteins. This is supported by observations in NSCLC where pharmacological reduction of BCL-2 expression promotes caspase-3/GSDME-mediated pyroptosis [68] (Fig. 3D).

The agreement between model predictions and independent experimental data confirms that the underlying regulatory dynamics are correctly captured, enabling us to explore how pyroptotic fate is determined. See Fig. 3 and Table S2 for more details.

### GSDMD and GSDME define orthogonal execution switches downstream of p53

To formally test whether the network can resolve the final choice between pyroptotic execution modes, we performed systematic gain- and loss-of-function perturbations of the pore-forming gasdermins GSDMD and GSDME under p53^K^ active, genotoxic stress conditions (Drug ON). These perturbations isolated the terminal decision while preserving upstream DNA damage signaling.

**Fig. 4.**
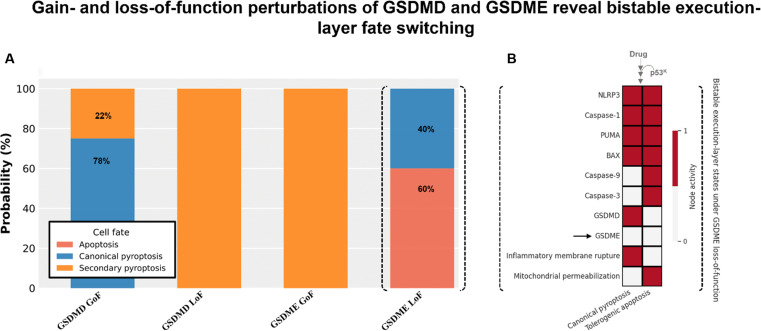
Gain- and loss-of-function perturbations reveal GSDMD and GSDME as orthogonal fate switches. (A) E1 denotes enforced activation [gain-of-function (GoF)], whereas KO denotes gene knockout [loss-of-function (LoF)]. GSDMD primarily controls canonical inflammasome-dependent pyroptosis, while GSDME stabilizes secondary pyroptotic execution and restricts apoptotic diversion upon loss. (B) Molecular activity patterns in the 2 coexisting attractors under GSDME KO, illustrating the divergence between canonical pyroptosis and apoptosis.

We first perturbed GSDMD, the canonical inflammasome-associated gasdermin. Enforced activation of GSDMD (E1) strongly biased the system toward canonical pyroptosis, which accounted for 78% of steady states, while a residual fraction (22%) resolved as secondary pyroptosis (Fig. 4). In contrast, GSDMD knockout (KO) completely eliminated canonical pyroptosis and redirected all trajectories toward secondary pyroptosis (100%), demonstrating that GSDMD is strictly required for inflammasome-driven execution but dispensable for caspase-3-dependent secondary membrane rupture.

We next examined GSDME, which is cleaved downstream of mitochondrial caspase activation. Enforced activation of GSDME (E1) collapsed executional heterogeneity and drove the system exclusively toward secondary pyroptosis (100%), abolishing both canonical pyroptosis and apoptotic outcomes (Fig. 4A). Strikingly, GSDME loss-of-function abolished stable secondary pyroptosis and generated bistability between apoptosis (60%) and canonical pyroptosis (40%), indicating that GSDME is required to stabilize the secondary pyroptotic attractor.

To resolve the molecular basis of this bistability, we analyzed terminal effector activities under GSDME KO (Fig. 4B). Both attractors retained active p53^K^ signaling, engaging inflammasome priming (NLRP3–caspase-1) and mitochondrial stress (PUMA–BAX). Fate commitment diverged at gasdermin–caspase activation: The canonical pyroptosis attractor showed caspase-1/GSDMD activity without caspase-9/3, whereas the apoptotic attractor activated caspase-9/3 while keeping GSDMD inactive. In both states, GSDME absence prevented caspase-3 from routing into secondary pyroptosis. Thus, GSDMD and GSDME are predicted to act as fate-committing switches downstream of shared p53^K^ signaling.

Notably, this predicted switch from secondary pyroptosis to apoptosis upon loss of GSDME function has been experimentally observed in lung adenocarcinoma cells, where MDM2 inhibition (restoring p53) reduced chemotherapy-induced GSDME-dependent pyroptosis and promoted apoptosis, supporting GSDME’s role as a key fate-switch node between immunogenic and tolerogenic cell death [69].

### Two interlocked feedback loops establish a bistable immunogenic switch between canonical and secondary pyroptosis

**Fig. 5:**
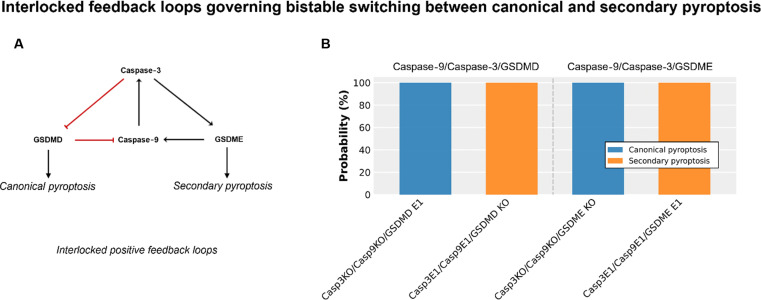
Interlocked feedback loops govern bistable switching between canonical and secondary pyroptosis. (A) Schematic representation of the 2 feedback loops identified in the network. (B) Monte Carlo simulation results (100,000 runs) demonstrating the functional consequence of perturbing each feedback loop. Coordinated gain-of-function (E1) or loss-of-function (KO) perturbations of the loop components produce all-or-nothing fate transitions, confirming that these motifs may function as robust fate-committing switches rather than passive downstream cascades.

Accordingly, our model predicts 2 interlocked, caspase-centered terminal decision feedback loops governing pyroptotic fate selection (Fig. 5A). The first is a double-negative (positive) feedback loop linking caspase-9, caspase-3, and GSDMD—a topology predicted to generate mutually exclusive switching between execution modes. The second is a positive feedback loop (caspase-9 → caspase-3 → GSDME) expected to reinforce commitment to secondary pyroptosis. All regulatory interactions closing these circuits are independently supported (Table S4).

To interrogate the functional consequences of these motifs, we performed systematic gain- and loss-of-function perturbations. Monte Carlo simulations (100,000 runs) of these perturbations demonstrate that the motifs function as robust fate-committing switches, producing all-or-nothing fate transitions (Fig. 5B). In the double-negative circuit, simultaneous knockout of caspase-9 and caspase-3 combined with enforced GSDMD activation (caspase-9 KO–caspase-3 KO–GSDMD E1) resulted in 100% canonical pyroptosis.Conversely, combined caspase-9/caspase-3 activation with GSDMD KO (caspase-9 E1–caspase-3 E1–GSDMD KO) enforced 100% secondary pyroptosis, confirming the loop’s switch-like function. Similarly, perturbation of the positive caspase-9–caspase-3–GSDME loop showed that its coordinated loss-of-function enforced canonical pyroptosis, while coordinated overactivation enforced uniform secondary pyroptosis.

To evaluate the robustness of these feedback circuits, we performed edge perturbation analysis by systematically deleting each directed interaction within the loops. While such perturbations are difficult to achieve experimentally, they are valuable in silico for testing the functionality and robustness of individual regulatory edges (Table 1). Deleting the inhibitory edge from GSDMD to caspase-9 abolished canonical pyroptosis. Deleting the activating edge from caspase-3 to GSDME eliminated secondary pyroptosis and caused an apoptotic attractor to emerge. Removing the remaining edges within these circuits left the attractor landscape unchanged, demonstrating that the network is robust to noncritical perturbations and that the predicted bistable switching behavior arises from the core feedback architecture rather than from specific modeling choices.

**Table 1.** Robustness analysis of feedback loops via targeted edge removal

Positive circuit	Removed interactions	Abrogated phenotypes
Caspase-9/caspase-3/GSDMD	Caspase-9/caspase-3	None
	Caspase-3/GSDMD	None
	GSDMD/caspase-9	Canonical pyroptosis
Caspase-9/caspase-3/GSDME	Caspase-9/caspase-3	None
	Caspase-3/GSDME	Secondary pyroptosis lost; apoptosis emerges
	GSDME/caspase-9	None

From a therapeutic perspective, this regulatory circuitry acts as an immunogenic fate switch: GSDMD dominance favors acute inflammatory canonical pyroptosis, whereas GSDME engagement redirects caspase signaling toward secondary pyroptosis with distinct, often less immunostimulatory, immune consequences.

### Clinical relevance of pyroptosis network nodes in NSCLC patient cohorts

To assess the clinical relevance of the modeled caspase–gasdermin execution axis, we analyzed the expression of a composite signature comprising *GSDMD*, *GSDME*, *NLRP3*, *CASP3*, and *CASP9* using GEPIA3 across NSCLC cohorts from TCGA. These nodes represent the minimal execution-layer decision module, encompassing inflammasome commitment (NLRP3), mitochondrial caspase signaling (*CASP9*–*CASP3*), and gasdermin-mediated membrane rupture (*GSDMD*/*GSDME*). Upstream sensors and obligate intermediates (e.g., caspase-1) were excluded to eliminate redundancy and maintain focus on fate-determining nodes.

**Fig. 6.**
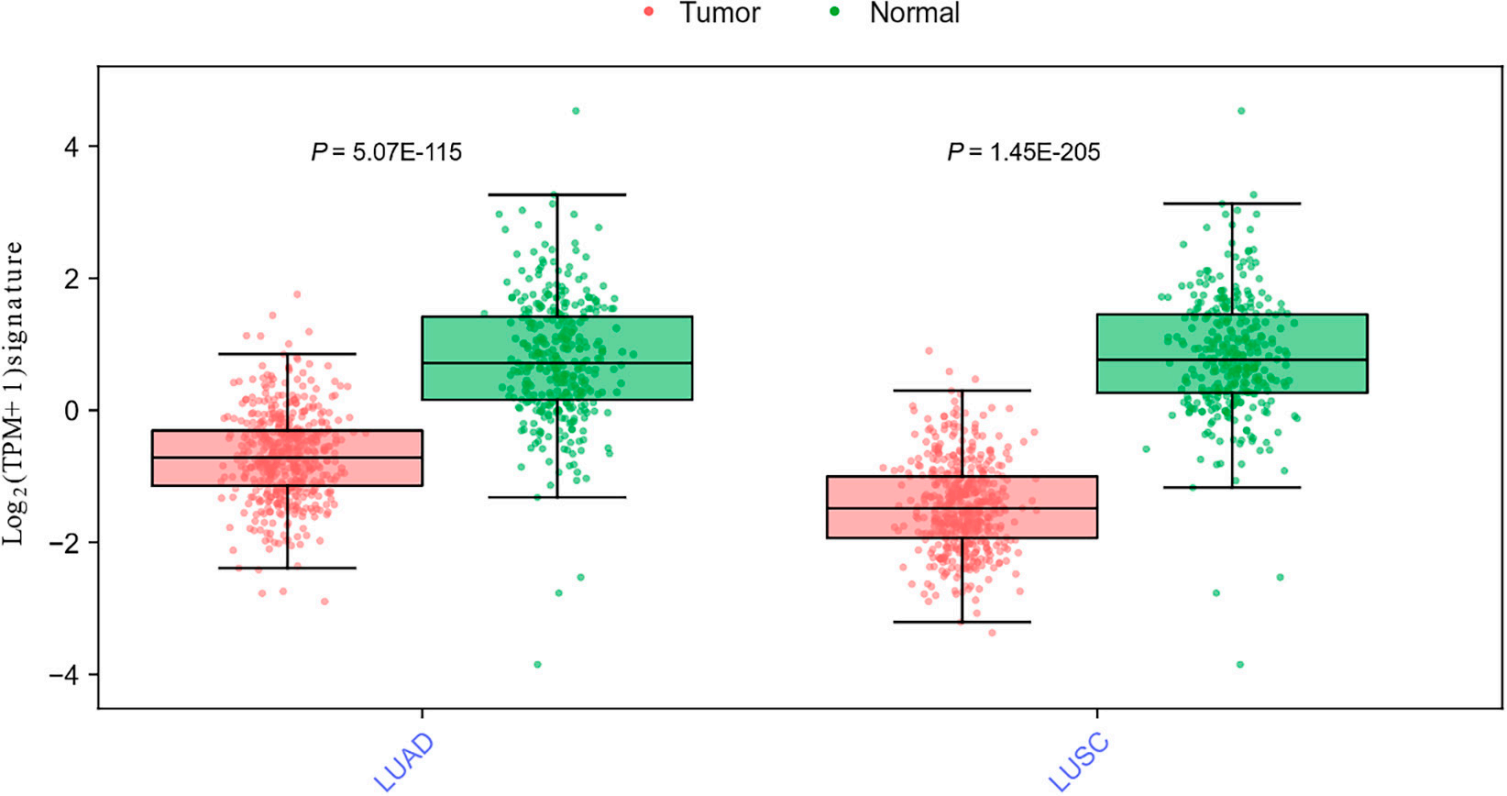
Expression of a composite pyroptosis execution-layer signature (*GSDMD*, *GSDME*, *NLRP3*, *CASP3*, *CASP9*) in LUAD and LUSC cohorts from TCGA. Boxplots show log_2_(TPM + 1) signature scores in tumor (red) and normal lung tissues (green), with individual samples overlaid as jittered points. Exact *P* values from differential expression analysis are shown above each comparison. Differential expression was assessed using GEPIA3’s limma-based pipeline with Benjamini–Hochberg FDR correction.

Strikingly, this execution-layer signature was significantly down-regulated in both LUAD and LUSC tumors compared with normal lung tissue (Fig. 6). In LUAD, the median signature score decreased from 0.72 in normal samples to −0.72 in tumors (*P* = 5.07 × 10^−115^), while in LUSC an even stronger repression was observed (−1.49 in tumors versus 0.76 in normals, *P* = 1.45 × 10^−205^). These results indicate a profound suppression of the pyroptotic execution machinery in NSCLC, consistent with a tumor-wide shift away from immunogenic cell death and supporting the model-predicted rewiring of caspase–gasdermin signaling toward non-inflammatory or tolerogenic outcomes.

The coordinated repression of both canonical and secondary pyroptosis executors suggests that NSCLC tumors actively constrain gasdermin-dependent death pathways, thereby stabilizing a non-immunogenic attractor state predicted by the network model.

Although TCGA data lack treatment annotations, the coordinated suppression of the pyroptosis execution-layer signature is expected to limit therapy-induced immunogenic cell death, providing a mechanistic basis for treatment non-immunogenic in NSCLC. Quantitative expression statistics for LUAD and LUSC cohorts are summarized in Table 2, with corresponding distributions shown in Fig. 6.

**Table 2.** GEPIA3-derived expression statistics for a composite pyroptosis execution-layer signature in NSCLC. Median signature scores [log_2_(TPM + 1)], sample numbers, *t* statistic (*t*), and *P* values from moderated *t* tests are shown for tumor versus normal lung tissues in lung adenocarcinoma (LUAD) and lung squamous cell carcinoma (LUSC) cohorts from TCGA. The composite signature comprises *GSDMD*, *GSDME*, *NLRP3*, *CASP3*, and *CASP9*. Differential expression was assessed using GEPIA3’s limma-based pipeline with Benjamini–Hochberg false discovery rate correction.

Name	Dataset	Tumor (*n*)	Normal (*n*)	Median tumor	Median normal	*t*	*P*
Signature	LUAD	515	347	−0.72	0.72	−26.724	5.07E−115
Signature	LUSC	498	338	−1.49	0.76	−41.589	1.45E−205

### Prognostic impact of pyroptosis execution-layer genes in NSCLC

To evaluate the prognostic relevance of the modeled pyroptosis execution layer, we performed a multivariable Cox proportional hazards analysis of core execution-layer genes using GEPIA3 across NSCLC cohorts. This approach assessed the independent contribution of each execution-layer node to overall survival within the modeled network context.

**Fig. 7.**
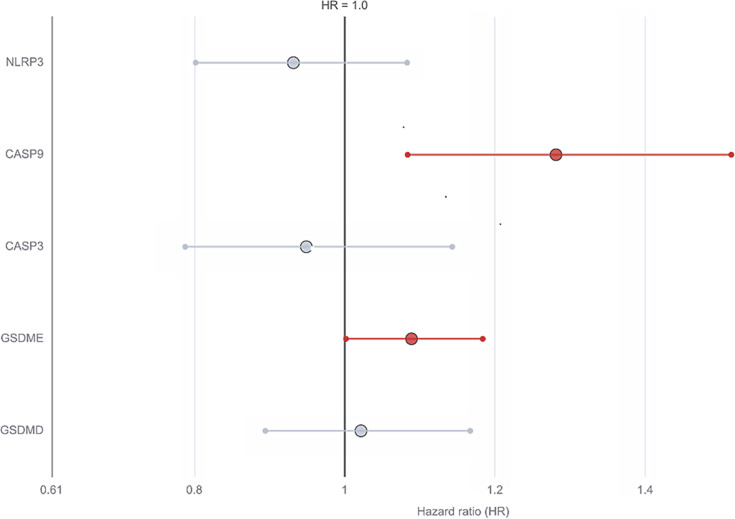
Multivariable Cox proportional hazards analysis of core pyroptosis execution-layer genes (*CASP3*, *CASP9*, *GSDMD*, *GSDME*, *NLRP3*) in NSCLC cohorts from TCGA, performed using GEPIA3’s survival module. Hazard ratios (HRs) with 95% confidence intervals indicate the independent association of each gene’s expression with overall survival.

Among the analyzed genes, *CASP9* and *GSDME* expression were significantly associated with poorer survival (Fig. 7). Elevated *CASP9* expression was associated with increased mortality risk (HR = 1.281, *P* = 0.00366), while *GSDME* showed a modest but significant adverse prognostic effect (HR = 1.089, *P* = 0.0449). In contrast, *CASP3*, *GSDMD*, and *NLRP3* did not exhibit significant survival associations, indicating that canonical pyroptosis executors are not independently prognostic in NSCLC.

These results are best interpreted in the context of 2 parallel, p53-regulated execution programs captured by the model. Canonical pyroptosis proceeds through the p53–NLRP3–CASP1–GSDMD axis and is independent of *CASP3* or *CASP9* activity, whereas secondary pyroptosis and apoptotic programs engage a distinct *p53–CASP9–CASP3–GSDME* module, uncoupled from inflammasome signaling. Crosstalk between these pathways enables flexible fate switching rather than linear execution. Accordingly, *CASP9* and *GSDME* emerge as clinically relevant fate-modulating nodes whose dysregulation biases cell death toward non-immunogenic outcomes, while canonical pyroptosis executors such as *GSDMD* lack independent prognostic value. These findings indicate that adverse clinical outcomes in NSCLC arise from qualitative rewiring of p53-driven death topology, rather than uniform suppression of the pyroptotic machinery (Fig. 7 and Table S5).

To further assess whether these associations depend on TP53 status, we stratified patients by TP53 mutation status. In the TP53-mutant subgroup, none of the 5 genes showed a statistically significant association with overall survival (all *P* > 0.05). In the TP53-WT subgroup, *CASP9* was significantly associated with worse overall survival (HR = 1.19, 95% CI: 1.06 to 1.34, *P* = 0.004). No significant associations were observed for *CASP3, GSDMD, GSDME*, or *NLRP3* in either TP53-stratified group (Table S6; see forest plot within as Fig. S1).

## Discussion

This study aimed to resolve how p53 activation coordinates the parallel canonical and secondary pyroptotic execution programs in NSCLC. Our dynamic Boolean model demonstrates that this coordination is governed by systems-level dynamics rather than linear signaling (Fig. 1). The network exhibits 3 stable, damage-induced states: senescence, canonical pyroptosis, and secondary pyroptosis (Fig. 2A and B). This inherent multistability provides a mechanistic explanation for the well-documented heterogeneity in therapeutic responses, where identical DNA-damaging agents can produce senescence, apoptosis, or inflammatory lysis in different cellular contexts [8,11,70]. Critically, the model shows that pyroptosis is not a default endpoint but a competitively stabilized outcome, reframing it from a downstream execution pathway into an emergent decision made at the downstream effector network.

Our findings support an emerging view of p53 as a systems-level coordinator rather than a simple apoptosis inducer. Beyond its established roles in cell-cycle arrest, senescence, autophagy, and ferroptosis [16,24,25,39], the model suggests that p53-driven pyroptosis in NSCLC is governed by a downstream execution module centered on caspase and gasdermin signaling, consistent with studies showing that p53 controls cell-fate programs through distinct network architectures. Thus, p53 functions as a master regulator of cell-fate plasticity, while the ultimate outcome is determined by downstream decision-making circuits. Here, the GSDMD/GSDME execution layer acts as the critical switch channeling p53 activation toward either immunogenic pyroptosis or apoptotic cell death.

### Validated model as a tool for mechanistic dissection

A key strength of the framework lies in its validation against experimentally established perturbation phenotypes (Fig. 3 and Table S2); the model shows excellent agreement with published observations across NSCLC systems. It recapitulates the finding that p53^K^ activation can prime both pyroptosis axes [8,9], that enforced caspase-3 or caspase-9 activity locks execution into secondary pyroptosis [49,67], and that BCL-2 overexpression suppresses lytic death [71]. This suppression is mechanistically supported by BCL-2’s recently identified direct inhibition of GSDMD cleavage [72]. This independent validation confirms the model captures the essential causal logic of the core decision module, making it a suitable tool for mechanistic dissection.

Crucially, validation simulations were performed under p53^K^-dominant conditions, isolating death-permissive dynamics and avoiding confounding by p53^A^-mediated senescence. This ensures that the model is interrogating execution control rather than stress sensing, supporting its suitability for mechanistic analysis of death-mode selection rather than pathway activation alone.

### Gasdermins determine how cancer cells die, not whether they die

One of the central unresolved questions in the field is why p53-induced death is highly inflammatory in some settings but tolerogenic in others. Our perturbation analysis provides a mechanistic answer by identifying GSDMD and GSDME as nonredundant execution stabilizers downstream of shared upstream signals (Fig. 4).

Consistent with experimental reports, enforced GSDMD activation locks cells into canonical inflammasome-dependent pyroptosis [73,74], whereas GSDME activation collapses executional heterogeneity and enforces secondary pyroptosis via caspase-3 cleavage [51,75]. Importantly, loss of GSDME does not abolish death signaling. Instead, it exposes bistability between canonical pyroptosis and apoptosis, despite persistent caspase-3 activity.

Our finding that GSDME loss diverts caspase-3 activity from secondary pyroptosis to apoptosis is not merely a computational prediction. This GSDME-dependent fate switch has been experimentally demonstrated in lung adenocarcinoma cells, where chemotherapy induces GSDME-mediated pyroptosis, but MDM2 inhibition (restoring p53 function) significantly reduces pyroptosis while increasing apoptosis [69]. This collective evidence, together with observations in other systems where GSDME licenses pyroptotic over apoptotic death [76] and operates as a parallel effector alongside GSDMD [77], indicates that the GSDME-mediated decision point is a conserved regulatory node that controls not only the mode of death but also the qualitative nature of the immune response across cell types, including epithelial cancers.

### How feedback loops enable discrete fate switching

Beyond individual nodes, we identified (in silico) 2 interlocked, caspase-centered motifs: a double-negative loop (caspase-9–caspase-3–GSDMD) that enables bistability, and a reinforcing loop (caspase-9–caspase-3cGSDME) that commits to secondary pyroptosis (Fig. 5). Feedback loops constitute the fundamental dynamical motifs responsible for generating multistability and stabilizing attractor states in Boolean regulatory networks. Therefore, analysis of these circuits provides mechanistic insight into how relatively small perturbations can reorganize global cell-fate decisions without requiring interpretation of the entire network topology. Systematic gain- and loss-of-function perturbations of these circuits produced all-or-nothing fate transitions, demonstrating that they function as active decision modules rather than passive downstream endpoints. This explains how modest changes in component activity, through mutation, expression changes, or pharmacological inhibition, can trigger abrupt, population-wide shifts in immunogenic output.

The reinforcing loop is supported by experimental evidence across multiple cancer types. In multiple myeloma, Liang et al. [78] demonstrated that N-GSDME itself penetrates the mitochondrial membrane to release cytochrome c and activate caspase-9/3, establishing a positive feedback circuit that amplifies pyroptotic commitment. Similar GSDME-mediated feedback has been observed in colorectal [75] and breast cancer [17], suggesting that this self-amplifying mechanism represents a conserved regulatory logic across malignancies. Given the conservation of these core pathway components in lung cancer, it is highly plausible that the same reinforcing GSDME feedback loop operates in NSCLC, where our model predicts it functions as a critical determinant of immunogenic versus tolerogenic cell fate.

### Clinical translation: Rewiring of the death-decision module in NSCLC tumors

Analysis of NSCLC patient cohorts adds a critical translational dimension. GEPIA3 analysis reveals coordinated down-regulation of the core execution module (*GSDMD*, *GSDME*, *NLRP3*, *CASP3*, *CASP9*) in tumors compared to normal tissue (Fig. 6 and Table 2). This suggests coordinated attenuation of key components of the pyroptotic execution machinery, consistent with the model framework. However, survival analysis reveals a critical nuance: High expression of *CASP9* and *GSDME*, but not canonical components, correlates with worse prognosis (Fig. 7). The absence of significant survival associations for *GSDMD*, *NLRP3*, and *CASP3* does not necessarily indicate limited biological importance. Rather, these molecules function within an interconnected regulatory network in which their biological effects depend on pathway topology, posttranslational activation, and cellular context, features that are not fully captured by transcript abundance alone.

It is important to note that these survival associations are correlational and do not imply causality, as TCGA data lack treatment information to determine whether expression patterns directly influence therapy response or reflect broader tumor biology. Furthermore, TP53-stratified analysis revealed that none of the execution-layer genes were significantly associated with overall survival in TP53-mutant tumors, whereas *CASP9* retained prognostic significance in the TP53-WT subgroup (Table S6 and Fig. S1). Although these findings remain associative, they are consistent with the model framework, which assumes intact p53 signaling and predicts that execution-layer dynamics are most relevant when upstream p53 activity is preserved.

Within the model framework, this pattern is highly informative. Canonical pyroptosis operates independently of *CASP9* and *CASP3*, whereas secondary pyroptosis and apoptosis require their activity. Thus, elevated *CASP9/GSDME* may reflect a partially retained execution module that enables stress-induced death while favoring secondary pyroptosis attractors. Whether these states differ in their immunological consequences remains to be experimentally determined. This is consistent with a model in which tumors may retain components of the death-execution machinery while altering the balance between alternative cell-death states, a hypothesis that warrants further experimental investigation [79,80].

### Why the pyroptosis switch matters therapeutically

Although both canonical and secondary pyroptosis are tumor-suppressive at the single-cell level, they differ profoundly in their immunological consequences. Canonical pyroptosis promotes robust cytokine release and immune activation, whereas secondary pyroptosis and apoptosis are generally considered less immunostimulatory [3,81,82]. However, emerging evidence demonstrates that GSDME-mediated pyroptosis can also elicit anti-tumor immunity in certain contexts. Peng et al. [83] showed that GSDME expression enhances cisplatin sensitivity in NSCLC by mediating pyroptosis and promoting T cell infiltration and chemokine release (MIP-1α, MIP-1β, MIP-2, IP-10) [83]. Similarly, Wu et al. [84] demonstrated that promoting GSDME-dependent pyroptosis enhances anti-tumor immune responses in vivo [84]. These findings indicate that the immunogenicity of secondary pyroptosis is context-dependent rather than strictly dichotomous [83,84].

Immunogenicity of pyroptosis likely exists along a spectrum rather than within a strict dichotomy, depending on factors such as gasdermin activation, the magnitude of inflammatory cytokine release, the tumor microenvironment, and the immune competence of the host. We acknowledge that the present study does not include direct analyses of immune responses or immune cell phenotypes, and therefore, our conclusions regarding immunogenic versus tolerogenic outcomes are based on model predictions and literature support. Consistent with this, Enssle et al. [82] recently demonstrated that GSDME links tumor cell-intrinsic nucleic acid signaling to proinflammatory cell death and successful checkpoint inhibitor immunotherapy [82]. Our model suggests a mechanism by which tumors could exploit differences between canonical and secondary pyroptosis. While the specific therapeutic implications extend beyond the current simulations, existing experimental evidence indicates several potential opportunities:

• In GSDMD-deficient tumors, enforcing GSDME-mediated secondary pyroptosis provides an alternative lytic route when apoptosis is resistant.
• In GSDME-silenced tumors, restoring or activating GSDMD is expected to mainly trigger highly immunogenic canonical pyroptosis.
  Thus, the evolutionary value of this switch, strategic flexibility for the cell, becomes a vulnerability. It may provide a fail-safe mechanism that preserves the potential for inflammatory cell death when one pyroptotic pathway is impaired, offering a clear rationale for gasdermin-targeted therapies. Our model suggests that therapeutic modulation of gasdermin expression or activation could reprogram tumor cell death toward more favorable immunological outcomes.

### The context-dependent role of GSDME across cancers

Our finding that high GSDME expression correlates with adverse prognosis in NSCLC appears paradoxical given its well-documented tumor-suppressive function in other malignancies. In gastric and colorectal cancers, for instance, GSDME is frequently silenced by promoter hypermethylation, and its restoration sensitizes cells to chemotherapy-induced pyroptosis [18,19]. Conversely, in other cancer models, GSDME expression can be up-regulated through transcriptional activation by p53 and Sp1, and its function varies with cellular context [85–87].

This context-dependent duality is consistent with what our model explains. GSDME does not act in isolation; its functional output depends on the configuration of the surrounding signaling network. When coupled with strong inflammasome activity (e.g., NLRP3–caspase-1–GSDMD), GSDME-mediated secondary pyroptosis can amplify immunogenic cell death. Importantly, recent work reveals that in NSCLC, GSDME also interacts physically with epidermal growth factor receptor (EGFR), stabilizing it and promoting extracellular signal-regulated kinase (ERK)-driven proliferation in the absence of genotoxic stress [88]. This pro-proliferative role likely underlies the adverse prognostic association we observed, positioning GSDME as a network-dependent node that can support tumor growth in inflammasome-attenuated contexts.

Thus, GSDME serves as a context-interpreting switch rather than a binary tumor suppressor or promoter. This systems-level perspective resolves apparent contradictions in the literature and suggests that GSDME-targeted interventions in NSCLC should be guided by co-assessment of EGFR and inflammasome signaling.

### Testable predictions and future directions

This reframing has direct experimental implications. The model generates several testable predictions:

1. CRISPR-mediated KO or inducible reexpression of GSDMD or GSDME should produce abrupt, switch-like changes in inflammatory output (e.g., IL-1β versus HMGB1 release) without altering upstream p53 activation.
2. Caspase-3 activation in GSDME-deficient cells should preferentially yield apoptotic [Annexin V^+^/propidium iodide (PI)^−^] rather than pyroptotic (PI rapid influx) morphology.
3. Pharmacological restoration of GSDMD activity in GSDME-low contexts should enhance cytokine release and immune recruitment following DNA damage.
  These predictions can be directly tested in p53 WT NSCLC models, where the NLRP3 inflammasome is a documented tumor-suppressive effector downstream of p53. Furthermore, the model’s general logic implies that the same fate-switching behavior may be observable in other cellular systems with analogous wiring, such as MCF7 breast cancer cells, where p53/PUMA/caspase-3/GSDME signaling is intact, even if NLRP3 is down-regulated. Validation across such models would distinguish context-specific pathway engagement from the conserved execution-layer decision principles our network reveals [89,90].
  To facilitate broader adoption of the framework by experimental and clinical researchers, we plan to develop an interactive web-based platform that will allow users to simulate perturbations of key regulatory nodes, visualize predicted attractor states, and explore cell-fate outcomes without requiring specialized expertise in Boolean network modeling. Such a resource would enhance reproducibility, enable external validation of model predictions, and provide a practical tool for generating hypothesis-driven experiments in diverse biological contexts. Detailed documentation and user guidelines will accompany future releases to maximize accessibility and translational utility.

### Limitations of the study

While our model provides a clear, logic-based framework for pyroptosis–apoptosis decision making in NSCLC, several inherent simplifications should be noted. The binary formalism captures core regulatory topology and attractor states but omits temporal kinetics, signaling gradients, and cellular heterogeneity. We focused on the p53-regulated axis due to its frequent alteration in NSCLC and its established role in linking DNA damage to inflammasome priming; therefore, p53-independent pyroptosis pathways (e.g., TLR-, AIM2-, or viral sensor-mediated) were excluded to maintain mechanistic focus, and their integration remains a direction for future work.

A key prediction of our model is that the interlocked feedback loops generate bistable switching between canonical and secondary pyroptosis. However, direct experimental evidence for hallmark bistable properties, such as hysteresis, irreversible state transitions, or coexistence of alternative stable states, is currently lacking in NSCLC systems. We therefore acknowledge that bistability remains a model prediction. Future studies employing time-lapse single-cell imaging of gasdermin activation, together with controlled pulse-stimulation or perturbation experiments, will be necessary to determine whether the predicted bistable behavior occurs in NSCLC cells.

Additionally, direct crosstalk between the GSDME and GSDMD pathways has not been fully established experimentally, and therefore, this aspect of the model remains a prediction requiring further validation.

The present model assumes WT p53 function, which is relevant to the subset of NSCLCs retaining functional p53 signaling. However, TP53 is mutated in approximately 50% of NSCLCs [7], with common hotspot mutations (e.g., R175H, R273H, and R248Q) exhibiting loss-of-function, dominant-negative, or gain-of-function activities. These mutations would likely alter network topology and attractor landscapes in several ways. Loss of DNA-binding capacity would impair transcriptional activation of PUMA, p21, and potentially NLRP3, thereby shifting the balance away from p53-dependent death outcomes. Gain-of-function mutants might engage alternative transcriptional programs or protein–protein interactions not captured in the current framework. Additionally, other common driver mutations in NSCLC, such as KRAS(G12C), EGFR(L858R), or STK11/LKB1 loss, could modulate the network through parallel pathways [e.g., AMPK signaling and RAS–mitogen-activated protein kinase (MAPK) crosstalk]. Systematic integration of these alterations represents an important future direction, potentially through mutation-specific parameterization or expansion of the network to include oncogenic signaling modules.

Transcriptomic validation focused on a downstream execution layer signature (*GSDMD*, *GSDME*, *NLRP3*, *CASP3*, *CASP9*) rather than upstream p53 targets, allowing the analysis to remain relevant across both TP53-WT and TP53-mutant contexts. The network does not explicitly incorporate immune modulatory or spatial microenvironmental signals, and TCGA data lack detailed treatment metadata, limiting therapy-specific inferences. Accordingly, all clinical correlations presented here should be interpreted as associative rather than causal. Additionally, the model integrates both transcriptional (e.g., p53 target gene expression) and posttranslational (e.g., caspase cleavage and GSDME activation) regulation using Boolean logic to approximate dynamic states. While TCGA/GTEx transcriptomic validation provides associative cohort-level support for predicted attractors, we acknowledge the absence of new preclinical functional experiments. Future studies will test key predictions in NSCLC models (e.g., siRNA knockdown of GSDME/CASP9 or p53 activation assays in A549/H460 lines) to confirm causality at the single-cell level.

**Fig. 8.**
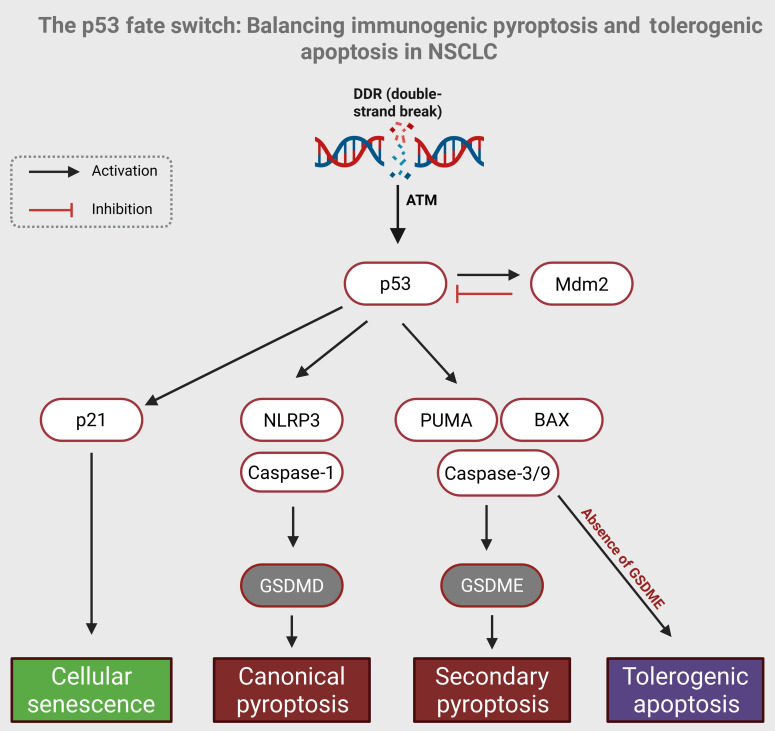
A p53-governed fate-switch network in NSCLC. Upon DNA double-strand break detection and ATM activation, p53 orchestrates a cell fate decision among 3 primary outcomes. p53 signaling diverges into 3 distinct regulatory pathways: (1) the induction of cellular senescence; (2) the activation of the canonical pyroptosis pathway via caspase-1 and GSDMD; and (3) the engagement of the mitochondrial pathway via PUMA, leading to caspase-9/3 activation. The output of this third arm is determined by GSDME availability, which acts as a molecular switch: GSDME activity routes signaling toward secondary pyroptosis, whereas its absence or inactivation redirects the same upstream signal toward tolerogenic apoptosis. This systems-level regulatory logic enables p53 to balance immunogenic and tolerogenic death outcomes alongside senescence. GSDME thus serves as the critical switch determining the immunogenic versus tolerogenic nature of cell death. In the schematic, black arrows represent activation and red hammerhead lines denote inhibition.

These limitations do not undermine the model’s mechanistic insights; instead, they highlight clear paths for refinement through single-cell multi-omics, dynamic perturbation data, and spatially resolved profiling. Nevertheless, this minimal model supports a framework in which p53 acts as the central orchestrator of the pyroptosis–apoptosis fate switch illustrated in Fig. 8.

In summary, this study establishes a systems-level framework in which p53-driven pyroptotic fate in NSCLC is governed not by upstream DNA damage sensing alone but by a terminal cell-death decision module embedded within the caspase–gasdermin axis. By integrating dynamic Boolean modeling, systematic perturbation analysis, and clinical transcriptomic data, our simulations indicate that canonical and secondary pyroptosis emerge as alternative stable attractors structured by this regulatory module, rather than linear signaling cascades. This reveals how NSCLC cells can switch between immunogenic pyroptosis and tolerogenic apoptosis under therapeutic stress. Within this framework, GSDMD functions as the indispensable executor of immunogenic canonical pyroptosis, whereas GSDME acts as a fate-modulating node that stabilizes secondary pyroptotic execution and constrains apoptotic diversion. Loss or dysregulation of GSDME exposes latent competition between mitochondrial and inflammasome-driven programs, enabling qualitative switching toward tolerogenic apoptotic outcomes without abolishing upstream p53 activity. This suggests that pyroptosis–apoptosis crosstalk is actively regulated at the terminal execution layer, not merely determined by initial damage intensity. Consistently, NSCLC tumors exhibit coordinated repression of this minimal pyroptosis decision module alongside selective adverse prognostic associations for *CASP9* and *GSDME*, indicating qualitative rewiring of terminal fate commitment rather than uniform loss of death competence. More broadly, this study highlights the topology of the terminal death-commitment network as a previously underappreciated determinant of cell-death immunogenicity, with direct implications for therapy-induced cell-death modulation in cancer.

## Data Availability

All data needed to evaluate the conclusions in the paper are present in the paper and/or the Supplementary Materials. The model code is available in the GitHub repository: https://github.com/gupatshan/Dynamic-model-for-pyroptosis-in-NSCLC, and is openly accessible for download and reuse.
