# Supplementary material for: p53 Orchestrates the Immunogenic-Tolerogenic Pyroptosis Switch in Non-Small Cell Lung Cancer: A Systems Biology Approach
Source: Comput Struct Biotechnol J. 2026 Jul 21;35(1):0172. doi: 10.34133/csbj.0172 (PMC13385539; doi:10.34133/csbj.0172)
Supplement: Supplementary 1 — Tables S1 to S6 [file csbj.0172.f1.zip › Table S1.pdf]

p53 Orchestrates the Immunogenic–Tolerogenic Pyroptosis Switch in Non–Small Cell Lung Cancer: A Systems Biology Approach

Author Information

Shantanu Gupta[1,*], Daner A. Silveira[2], Rodrigo Juliani Siqueira Dalmolin[1], José Carlos M. Mombach[3], and Ronaldo F. Hashimoto[4]

Affiliations

[1] Bioinformatics Multidisciplinary Environment-BioME – Digital Metropole Institute, Federal University of Rio Grande do Norte, Natal 59076550, RN, Brazil

[2] Children's Cancer Institute, Porto Alegre, Rio Grande do Sul, Brazil

[3] Departamento de Física, Universidade Federal de Santa Maria, Santa Maria 97105-900, RS, Brazil

[4] Instituto de Matemática e Estatística, Departamento de Ciência da Computação, Universidade de São Paulo, Rua do Matão 1010, 05508-090, São Paulo - SP, Brasil

Corresponding author:

*Corresponding to: Shantanu Gupta (S.G), https://orcid.org/0000-0001-7110-6564; Email: shantanu.gupta@imd.ufrn.br ;

Table S1

Table S1: Logical Rules Governing Node States in the Model (Highlighted in cream colour) Illustrated in Fig. 1 (main paper), Depicting the State of Each Node Based on Its Regulators. The left-hand side presents the official names of the molecules (target nodes) (highlighted in Gray), while the right-hand side lists the references of each interaction leading to the target node.

Official names of the molecules	Target Node	No. Interactions	Interaction	Biological Justification	References
**ATM serine/threonine kinase**	ATM	1	Drug	DNA damage activates ATM.	PMID: 29620203
		2	Wip1	Phosphatase Wip1 deactivates ATM	PMID: 16949371
		3	E2F1	E2F1 promotes ATM expression/activity.	PMID: 11459832
		Rule	Drug AND (E2F1 OR NOT Wip1)		ATM activation occurs in the presence of DNA damage (Drug) when either

					E2F1 is active or Wip1 is inactive.
**AMP-activated Protein Kinase**	AMPK	4	Drug	DNA damage-induced energy stress activates AMPK.	PMID: 33824293
		5	ATM	ATM phosphorylates/activates AMPK.	PMID: 33824293
		Rule	Drug AND ATM		AMPK activation requires both DNA damage (Drug) and active ATM.
**Mouse Double Minute 2 (E3 ubiquitin ligase)**	Mdm2	6	ATM	ATM phosphorylates and inhibits Mdm2, preventing p53 degradation.	PMID: 27568562
		7	P53_A	p53_A transcriptionally activates Mdm2 as part of negative feedback.	PMID: 25672611
		8	AKT	AKT phosphorylates and stabilizes Mdm2, promoting p53 degradation.	PMID: 33880579
		Rule	NOT ATM OR p53_A AND AKT		Mdm2 is active either in the absence of ATM, or

					when both p53_A and AKT are present.
**Wild-type p53-induced Phosphatase 1 (PPM1D)**	Wip1	9	E2F1	E2F1 transcriptionally activates Wip1 expression.	PMID: 22201816
		Rule	E2F1		Wip1 can be activated when E2F1 is present.
**AKT Serine/Threonine Kinase (Protein Kinase B)**	AKT	10	AMPK	AKT is active when AMPK is absent.	PMID: 33824293
		Rule	NOT AMPK		AKT is activated if and only if AMPK is absent.
**$p53^{A}$ (Arrest)**	p53_A	11	P53_K	p53_K and p53_A are mutually exclusive functional forms.	PMID: 21576488
		12	ATM	ATM phosphorylates/stabilizes p53_A under DNA damage.	PMID: 27612029
		13	AMPK	AMPK promotes p53_A activity under metabolic stress.	PMID: 24190973
		14	P53_INP1	p53INP1 favors p53_K; its absence allows p53_A activity.	PMID: 34718338

		15	Mdm2	Mdm2 promotes p53_A degradation.	PMID: 19448627
		16	Wip1	Wip1 dephosphorylates/inactivates p53_A.	PMID: 27959454
		Rule	NOT p53_K AND ATM AND (AMPK OR NOT p53INP1) AND NOT Mdm2AND NOT Wip1		p53_A is stabilized under DNA damage (ATM ON) when Mdm2 and Wip1 are suppressed, and either metabolic stress (AMPK ON) or low p53INP1 promotes arrest over death signaling.
**p53-Inducible Nuclear Protein 1**	p53INP_1	17	p53_A	Activated by both p53_A and p53_K as a stress-responsive regulator.	PMID: 34718338
		18	p53_K	Activated by both p53_A and p53_K as a stress-responsive regulator.	PMID: 34718338
		Rule	p53_A OR p53_A		p53INP1 is activated when either p53_A or p53_K is active.
**$p53^K$ (Killer)**	p53_K	19	p53_A	p53_A inhibits p53_K activation — the two p53	PMID: 21576488

				forms are mutually exclusive.	
		20	ATM	Phosphorylates/stabilizes p53_K under DNA damage.	PMID: 27612029
		21	Mdm2	Promotes degradation of p53_K; p53_K is active when Mdm2 is absent.	PMID: 19448627
		Rule	NOT p53_A AND ATM AND NOT Mdm2		p53_K is activated when p53_A is inactive, ATM is active, and Mdm2 is inactive.
**p21 (CDKN1A, Cyclin-dependent kinase inhibitor 1)**	p21	22	P53_A	Transactivates p21 expression, promoting cell cycle arrest.	PMID: 11892838
		23	Caspase 3	p21 inhibits Caspase-3 activity to block apoptosis.	PMID: 39706988
		24	AKT	p21 is suppressed when AKT is active via survival signaling.	PMID: 36522339
		Rule	p53_A OR (NOTCaspase3 AND NOTAKT)		p21 is activated when either p53_A is active or when both Caspase-3 and AKT

					are inactive.
**NLR Family Pyrin Domain Containing 3 (inflammasome)**	NLRP3	25	P53_K	Transcriptional activation of NLRP3 inflammasome components.	PMID: 36653338
		26	BCL2	Bcl2 Inhibits NLRP3 inflammasome assembly	PMID: 22342844
		Rule	P53_K AND NOT BCL2		NLRP3 is activated when p53_K is active and BCL2 is inactive.
**p53 Upregulated Modulator of Apoptosis (BBC3)**	PUMA	27	P53_K	Transcriptional activation of PUMA, promoting mitochondrial apoptosis.	PMID: 16675590
		Rule	P53_K		PUMA is activated when p53_K is active.
**BCL2-Associated X Protein**	BAX	28	P53_K	Transcriptional activation of BAX.	PMID: 16675590
		29	BCL2	BCL2 binds and inhibits BAX	PMID: 12581565
		Rule	P53_K AND NOT BCL2		BAX is activated when p53_K is active and BCL2 is inactive.
**Cysteine-aspartic protease 1 (inflammasome caspase)**	Caspase 1	30	NLRP3	NLRP3 inflammasome activates Caspase-1	PMID: 30365491
		Rule	NLRP3		Caspase 1 is activated

					when NLRP3 is active.
**Cysteine-aspartic protease 3 (executioner caspase)**	Caspase 3	31	BAX	Promotes mitochondrial outer membrane permeabilization, leading to Caspase-9 activation.	PMID: 30365491
		32	Caspase 9	Cleaves and activates Caspase-3.	PMID: 30365491
		33	BCL2	BCL2 Inhibits Caspase 3 expression.	PMID: 16297711
		34	Caspase 1	Inflammasome-activated Caspase-1 directly cleaves Caspase-3.	PMID: 31064994
		35	p21	Binds and inhibits Caspase-3 activity.	PMID: 39706988
		Rule	(BAX AND Caspase9) OR (NOT BCL2 AND Caspase1 AND NOT p21)		Caspase-3 is activated through the mitochondrial pathway (BAX and Caspase-9) or the inflammasome pathway (Caspase-1) when BCL2 is absent and p21 is not active.
**B-cell Lymphoma 2 (anti-apoptotic protein)**	BCL2	36	PUMA	BCL2 is inactive when PUMA is present.	PMID: 27650927

		37	AKT	Phosphorylates and stabilizes BCL2, promoting cell survival.	PMID: 19139118
		Rule	NOT PUMA OR AKT		BCL2 is active when either PUMA is absent or AKT is present.
**Cell Division Cycle 25 (phosphatase)**	Cdc25		ATM	ATM Phosphorylates and inhibits Cdc25 under DNA damage.	PMID: 19060337
		Rule	NOT ATM		Cdc25 is active when ATM is absent.
**Cyclin-dependent kinase – Cyclin complex**	CdkCyclin complex	38	p21	P21 Binds and inhibits Cdk-Cyclin activity.	PMID: 7626805
		39	Cdc25	Cdc25 activates Cdk-Cyclin by dephosphorylation.	PMID: 11805096
		Rule	NOT p21 AND Cdc25		CdkCyclin is active when p21 is absent and Cdc25 is present.
**Gasdermin D**	GSDMD	40	Caspase 1	Caspase 1, cleaves GSDMD to induce pyroptosis.	PMID: 38214430
		41	Caspase 3	Caspase 3 Inhibits GSDMD cleavage when active.	PMID: 30106450

		42	AMPK	AMPK Energy stress via AMPK suppresses GSDMD activation.	PMID: 37495617
		Rule	Caspase1 AND (NOT Caspase3 OR NOT AMPK)		GSDMD is cleaved when Caspase-1 is active and either Caspase-3 or AMPK is inactive.
**Cysteine-aspartic protease 9 (initiator caspase, mitochondrial)**	Caspase 9	43	GSDME	Cleaved GSDME promotes Caspase-9 activation.	PMID: 34553845
		44	BCL2	Inhibits mitochondrial apoptosis	PMID: 29927992
		45	GSDMD	Canonical pyroptosis via GSDMD inhibits Caspase-9 activity.	PMID: 31064994
		Rule	GSDME OR NOT(BCL2 OR GSDMD)		Caspase-9 is activated when GSDME is present, or when both BCL2 and GSDMD are absent.
**Gasdermin E**	GSDME	46	Caspase 3	Caspase 3 Cleaves GSDME to induce secondary pyroptosis.	PMID: 32839451
		47	AMPK	AMPK-mediated energy stress promotes GSDME cleavage.	PMID: 37460805

		Rule	Caspase3 AND AMPK		GSDME is cleaved when both Caspase-3 and AMPK are active.
**E2F Transcription Factor 1**	E2F1	48	RB1	RB1 Binds and inhibits E2F1.	PMID: 15016799
		49	Cdc25	Cdc25 Activates cyclin-CDK, which phosphorylates RB1, releasing E2F1.	PMID: 10454584
		Rule	NOT RB1 AND Cdc25		E2F1 is active when RB1 is inactive and Cdc25 is active.
**Retinoblastoma Protein 1**	RB1	50	CDKCyclin	CDK-Cyclin Phosphorylates RB1, inactivating it and releasing E2F1.	PMID: 36399634
		Rule	NOT CdkCyclin		RB1 is active when the CDK-Cyclin complex is inactive.
**Cellular senescence phenotype**	Senescence	51	P21	P21 Induces cell cycle arrest and senescence.	PMID: 17515610
		52	P53_A	Transcriptional activation of p21, promoting senescence.	PMID: 17515610
		Rule	p21 AND p53_A		Senescence occurs when both p21 and

					p53_A are active.
**Gasdermin D-dependent pyroptosis**	Canonical pyroptosis	53	GSDMD	GSDMD, Cleaved by Caspase-1 to form plasma membrane pores, executing canonical pyroptosis.	PMID: 40894439
		Rule	GSDMD		Canonical pyroptosis occurs when GSDMD is active.
**Gasdermin E-dependent pyroptosis**	Secondary Pyroptosis	54	GSDME	GSDME, Cleaved by Caspase-3 to form membrane pores, executing secondary pyroptosis.	PMID: 38104141
		Rule	GSDME		Secondary Pyroptosis occurs when GSDME is active.
**Cell proliferation phenotype**	Proliferation	55	BCL2	BCL2, promotes cell survival and proliferation by inhibiting apoptosis.	PMID: 40136517
		56	E2F1	BCL2 and E2F1 support active cell proliferation.	PMID: 40437556
		Rule	BCL2 AND E2F1		Proliferation occurs when both BCL2 and E2F1 are active.
**Tolerogenic Apoptosis**		57	Caspase 3	Apoptosis is activated	PMID: 10733772

				when Caspase-3 is active and G SDME is inactive.	
	**Tolerogenic Apoptosis**	58	GSDME	**Note on the GSDME–Apoptosis rule:** The logical link GSDME inhibits Apoptosis does not imply direct biochemical inhibition. Rather, it captures the experimentally established competitive routing of caspase-3 activity: when GSDME is present and cleaved by caspase-3, cells execute pyroptosis; when GSDME is absent, caspase-3 engages apoptotic effectors. This rule ensures mutual exclusivity between pyroptotic and apoptotic fates, consistent with observations that GSDME loss redirects caspase-3-mediated death from pyroptosis to apoptosis (PMID: 28459430, PMID: 33133646, PMID: 35002520)	
		Rule	Caspase 3 AND NOT GSDME		
