# Supplementary material for: p53 Orchestrates the Immunogenic-Tolerogenic Pyroptosis Switch in Non-Small Cell Lung Cancer: A Systems Biology Approach
Source: Comput Struct Biotechnol J. 2026 Jul 21;35(1):0172. doi: 10.34133/csbj.0172 (PMC13385539; doi:10.34133/csbj.0172)
Supplement: Supplementary 1 — Tables S1 to S6 [file csbj.0172.f1.zip › Table S2.pdf]

p53 Orchestrates the Immunogenic–Tolerogenic Pyroptosis Switch in Non–Small Cell Lung Cancer: A Systems Biology Approach

Author Information

Shantanu Gupta[1,*], Daner A. Silveira[2], Rodrigo Juliani Siqueira Dalmolin[1], José Carlos M. Mombach[3], and Ronaldo F. Hashimoto[4]

Affiliations

[1] Bioinformatics Multidisciplinary Environment-BioME – Digital Metropole Institute, Federal University of Rio Grande do Norte, Natal 59076550, RN, Brazil

[2] Children's Cancer Institute, Porto Alegre, Rio Grande do Sul, Brazil

[3] Departamento de Física, Universidade Federal de Santa Maria, Santa Maria 97105-900, RS, Brazil

[4] Instituto de Matemática e Estatística, Departamento de Ciência da Computação, Universidade de São Paulo, Rua do Matão 1010, 05508-090, São Paulo - SP, Brasil

Corresponding author:

*Corresponding to: Shantanu Gupta (S.G), https://orcid.org/0000-0001-7110-6564; Email: shantanu.gupta@imd.ufrn.br ;

Table S2

Table S2: Validation of model predictions against Confirmed Experimental Observations. Comparison of simulated Gain-of-Function (GoF) perturbations for key nodes (p53_K, Caspase-1, Caspase-9, BCL-2) with established experimental outcomes across multiple cell lines, including NSCLC.

**Consistency Between the Model Predictions and Confirmed Experimental Observations**				
**Perturbation**	**Model Prediction**	**Experimental Observation**	**Cell Lines**	**Reference**
p53_K E1	Coexistence of canonical and secondary pyroptosis.	p53 induces NLRP3/caspase-1/GSDMD and caspase-3/GSDME cleavage.	293T, SH-SY5Y, HCC1937, T-47D, MDA-MB-231, MDA-MB-468, HepG2, and A549	[1,2]
Caspase-3 E1	100% secondary pyroptosis (GSDME activation).	Caspase-3 cleaves GSDME → pyroptotic lysis.	NCI-H226, SK-MES-1, A549, NCI-HCC827, NCI-H1975, PC9 and BEAS-2B cells	[3]

Caspase-9 E1	Secondary pyroptosis via caspase-3/GSDME.	Mitochondrial caspase-9 activates caspase-3 → GSDME.	Human A549 and H1299 cell lines	[4]
BCL-2 E1	Suppression of pyroptosis; promotion of survival/drug resistance phenotype.	Pharmacological BCL-2 reduction promotes pyroptosis (consistent with anti-pyroptotic role)	A549	[5]

References:

[1] T. Zhang, Y. Li, R. Zhu, P. Song, Y. Wei, T. Liang, G. Xu, Transcription Factor p53 Suppresses Tumor Growth by Prompting Pyroptosis in Non-Small-Cell Lung Cancer, Oxid Med Cell Longev 2019 (2019) 8746895. https://doi.org/10.1155/2019/8746895.

[2] J. Wang, Y. Wang, H. Xiao, W. Yang, W. Zuo, Z. You, C. Wu, J. Bao, Dynamic O-GlcNAcylation coordinates etoposide-triggered tumor cell pyroptosis by regulating p53 stability, Journal of Biological Chemistry 301 (2025) 108050. https://doi.org/10.1016/j.jbc.2024.108050.

[3] F. Yu, W. Tan, Z. Chen, X. Shen, X. Mo, X. Mo, J. He, Z. Deng, J. Wang, Z. Luo, J. Yang, Nitidine chloride induces caspase 3/GSDME-dependent pyroptosis by inhibting PI3K/Akt pathway in lung cancer, Chin Med 17 (2022) 115. https://doi.org/10.1186/s13020-022-00671-y.

[4] Y. Gao, X. Zhai, C. Zhang, H. Zhao, B. Ji, R. Sun, X. Du, Y. Du, S. Gao, Y. Zhang, T. Wang, Fosinopril mediates antitumor efficacy by inducing GSDME-dependent pyroptosis in NSCLC, Cell Death Discov. 11 (2025) 540. https://doi.org/10.1038/s41420-025-02791-4.

[5] Z. Hu, Y. Lai, C. Ma, L. Zuo, G. Xiao, H. Gao, B. Xie, X. Huang, H. Gan, D. Huang, N. Yao, B. Feng, J. Ru, Y. Chen, D. Cai, Cordyceps militaris extract induces apoptosis and pyroptosis via caspase-3/PARP/GSDME pathways in A549 cell line, Food Science & Nutrition 10 (2022) 21–38. https://doi.org/10.1002/fsn3.2636.
