# Supplementary material for: p53 Orchestrates the Immunogenic-Tolerogenic Pyroptosis Switch in Non-Small Cell Lung Cancer: A Systems Biology Approach
Source: Comput Struct Biotechnol J. 2026 Jul 21;35(1):0172. doi: 10.34133/csbj.0172 (PMC13385539; doi:10.34133/csbj.0172)
Supplement: Supplementary 1 — Tables S1 to S6 [file csbj.0172.f1.zip › Table S3.pdf]

p53 Orchestrates the Immunogenic–Tolerogenic Pyroptosis Switch in Non–Small Cell Lung Cancer: A Systems Biology Approach

Author Information

Shantanu Gupta[1,*], Daner A. Silveira[2], Rodrigo Juliani Siqueira Dalmolin[1], José Carlos M. Mombach[3], and Ronaldo F. Hashimoto[4]

Affiliations

[1] Bioinformatics Multidisciplinary Environment-BioME – Digital Metropole Institute, Federal University of Rio Grande do Norte, Natal 59076550, RN, Brazil

[2] Children's Cancer Institute, Porto Alegre, Rio Grande do Sul, Brazil

[3] Departamento de Física, Universidade Federal de Santa Maria, Santa Maria 97105-900, RS, Brazil

[4] Instituto de Matemática e Estatística, Departamento de Ciência da Computação, Universidade de São Paulo, Rua do Matão 1010, 05508-090, São Paulo - SP, Brasil

Corresponding author:

*Corresponding to: Shantanu Gupta (S.G), https://orcid.org/0000-0001-7110-6564; Email: shantanu.gupta@imd.ufrn.br ;

Table S3

Table S3. Independent experimental validation of model predictions with transparency of interaction sources. This table compares model predictions with experimental observations from studies that were not used during network construction, thereby providing an independent test of model accuracy. For each perturbation scenario, the predicted pathway and resulting phenotype are shown alongside the corresponding experimental finding and its source (Validation Study). The "Interaction Source" column cites the primary literature that originally established each regulatory edge in the model (e.g., p53 → NLRP3, Casp3 → GSDME), offering transparency regarding the evidence base for network topology while clearly distinguishing it from validation studies. Agreement between model prediction and experimental observation is indicated as "Yes" when the simulated outcome matches the reported phenotype. Pathway arrows (→) denote activation, whereas (⊣) indicates inhibition.

Model Prediction (with Pathway)	Experimental Observation	Validation Study	Interaction Source (for transparency)	Agreement
p53 Overexpression → Canonical Pyroptosis (p53 → NLRP3 → Casp1 → GSDMD)	p53 stabilization triggers NLRP3/GSDMD pyroptosis in NSCLC	Zhang et al. 2019 [1] (PMID: 31737176)	NLRP3 → Casp1, Lasithiotaki et al. 2018 [2] (PMID: 30365491); Casp1 → GSDMD, Zhao et al. 2024 [3] (PMID: 38214430)	Yes
p53 Overexpression → Secondary Pyroptosis (p53 → PUMA → Bax →	p53-dependent GSDME cleavage in NSCLC cells	Wang et al. 2025 [4] (PMID: 39667498)	p53 → PUMA → Bax → Casp9/Casp3, Yu et al. 2006 [5] (PMID: 16675590); Casp3 → GSDME, Peng	Yes

Casp9/Casp3 → GSDME)			et al. 2020 [6] (PMID: 32839451)	
p53 Overexpression → Secondary Pyroptosis (p53 → PUMA → Casp9 → Casp3 → GSDME)	Caspase-3 activates GSDME in NSCLC cells	Yu et al. 2022 [7] (PMID: 36175965)	p53 → PUMA → Bax → Casp9/Casp3, Yu et al. 2006 [5] (PMID: 16675590); Casp3 → GSDME, Peng et al. 2020 [6] (PMID: 32839451)	Yes
p53 + Casp9 Overexpression → Secondary Pyroptosis (Casp9 → Casp3 → GSDME)	Casp9 activation triggers GSDME pyroptosis in NSCLC cells	Gao et al. 2025 [8] (PMID: 41271670)	Casp9 → Casp3, Yu et al. 2006 [5] (PMID: 16675590); Casp3 → GSDME, Peng et al. 2020 [6] (PMID: 32839451)	Yes
BCL2 Overexpression → Pyroptosis Suppressed (BCL2 inhibits NLRP3 and GSDMD)	BCL-2 blocks pyroptosis in NSCLC cells	Hu et al. 2022 [9] (PMID: 35035907)	BCL2 ⊣ NLRP3, Shimada et al. 2012 [10] (PMID: 22342844) ; BCL2 ⊣ Casp3, Lin et al. 2005 [11] (PMID: 16297711)	Yes

References:

1 Zhang T, Li Y, Zhu R, Song P, Wei Y, Liang T & Xu G (2019) Transcription Factor p53 Suppresses Tumor Growth by Prompting Pyroptosis in Non-Small-Cell Lung Cancer. *Oxid Med Cell Longev* **2019**, 8746895.

2 Lasithiotaki I, Tsitoura E, Samara KD, Trachalaki A, Charalambous I, Tzanakis N & Antoniou KM (2018) NLRP3/Caspase-1 inflammasome activation is decreased in alveolar macrophages in patients with lung cancer. *PLoS One* **13**, e0205242.

3 Zhao X, Chen C, Han W, Liang M, Cheng Y, Chen Y, Pang D, Lei H, Feng X, Cao S, Li Z, Wang J, Zhang Y & Yang B (2024) EEBR induces Caspase-1-dependent pyroptosis through the NF-κB/NLRP3 signalling cascade in non-small cell lung cancer. *J Cell Mol Med* **28**, e18094.

4 Wang J, Wang Y, Xiao H, Yang W, Zuo W, You Z, Wu C & Bao J (2025) Dynamic O-GlcNAcylation coordinates etoposide-triggered tumor cell pyroptosis by regulating p53 stability. *Journal of Biological Chemistry* **301**, 108050.

5 Yu J, Yue W, Wu B & Zhang L (2006) PUMA sensitizes lung cancer cells to chemotherapeutic agents and irradiation. *Clin Cancer Res* **12**, 2928–2936.

6 Peng Z, Wang P, Song W, Yao Q, Li Y, Liu L, Li Y & Zhou S (2020) GSDME enhances Cisplatin sensitivity to regress non-small cell lung carcinoma by mediating pyroptosis to trigger antitumor immunocyte infiltration. *Signal Transduct Target Ther* **5**, 159.

7 Yu F, Tan W, Chen Z, Shen X, Mo X, Mo X, He J, Deng Z, Wang J, Luo Z & Yang J (2022) Nitidine chloride induces caspase 3/GSDME-dependent pyroptosis by inhibting PI3K/Akt pathway in lung cancer. *Chin Med* **17**, 115.

8 Gao Y, Zhai X, Zhang C, Zhao H, Ji B, Sun R, Du X, Du Y, Gao S, Zhang Y & Wang T (2025) Fosinopril mediates antitumor efficacy by inducing GSDME-dependent pyroptosis in NSCLC. *Cell Death Discov* **11**, 540.

9 Hu Z, Lai Y, Ma C, Zuo L, Xiao G, Gao H, Xie B, Huang X, Gan H, Huang D, Yao N, Feng B, Ru J, Chen Y & Cai D (2022) Cordyceps militaris extract induces apoptosis and pyroptosis via caspase-3/PARP/GSDME pathways in A549 cell line. *Food Science & Nutrition* **10**, 21–38.

10 Shimada K, Crother TR, Karlin J, Dagvadorj J, Chiba N, Chen S, Ramanujan VK, Wolf AJ, Vergnes L, Ojcius DM, Rentsendorj A, Vargas M, Guerrero C, Wang Y, Fitzgerald KA, Underhill DM, Town T & Arditi M (2012) Oxidized Mitochondrial DNA Activates the NLRP3 Inflammasome during Apoptosis. *Immunity* **36**, 401–414.

11 Lin H-I, Lee Y-J, Chen B-F, Tsai M-C, Lu J-L, Chou C-J & Jow G-M (2005) Involvement of Bcl-2 family, cytochrome *c* and caspase 3 in induction of apoptosis by beauvericin in human non-small cell lung cancer cells. *Cancer Letters* **230**, 248–259.
