# Supplementary material for: p53 Orchestrates the Immunogenic-Tolerogenic Pyroptosis Switch in Non-Small Cell Lung Cancer: A Systems Biology Approach
Source: Comput Struct Biotechnol J. 2026 Jul 21;35(1):0172. doi: 10.34133/csbj.0172 (PMC13385539; doi:10.34133/csbj.0172)
Supplement: Supplementary 1 — Tables S1 to S6 [file csbj.0172.f1.zip › Table S4.pdf]

p53 Orchestrates the Immunogenic–Tolerogenic Pyroptosis Switch in Non–Small Cell Lung Cancer: A Systems Biology Approach

Author Information

Shantanu Gupta[1, *], Daner A. Silveira[2], Rodrigo Juliani Siqueira Dalmolin[1], José Carlos M. Mombach[3], and Ronaldo F. Hashimoto[4]

Affiliations

[1] Bioinformatics Multidisciplinary Environment-BioME – Digital Metropole Institute, Federal University of Rio Grande do Norte, Natal 59076550, RN, Brazil

[2] Children's Cancer Institute, Porto Alegre, Rio Grande do Sul, Brazil

[3] Departamento de Física, Universidade Federal de Santa Maria, Santa Maria 97105-900, RS, Brazil

[4] Instituto de Matemática e Estatística, Departamento de Ciência da Computação, Universidade de São Paulo, Rua do Matão 1010, 05508-090, São Paulo - SP, Brasil

Corresponding author:

[*]Corresponding to: Shantanu Gupta (S.G), https://orcid.org/0000-0001-7110-6564; Email: shantanu.gupta@imd.ufrn.br ;

Table S4

Table S4: Experimental observations and mechanisms supporting the Caspase-3–GSDMD–Caspase-9 and Caspase-3–GSDME–Caspase-9 feedback loops.

Positive feedback loop	Circuit elements	Targets	Interaction type	Mechanism	Ref.
Double Negative (positive) Feedback Loop					
Caspase-3/GSDMD/Caspase-9	Caspase-3	GSDMD	Direct Inhibition	Caspase-3 cleaves and inactivates GSDMD, preventing GSDMD pore formation and pyroptosis execution.	[1]
	GSDMD	Caspase-9	Direct Inhibition	GSDMD inhibits caspase-9 activation, as caspase-1-induced apoptosis proceeds via caspase-9 in GSDMD-deficient cells.	[2]
	Caspase-9	Caspase-3	Direct Activation	Caspase-9 cleaves and activates Caspase-3 as part of the intrinsic apoptosis pathway.	[3]
Positive Feedback Loop					
Caspase-3/GSDME/Caspase-9	Caspase-3	GSDME	Direct Activation	Caspase-3 cleaves GSDME to release its N-terminal pore-forming domain, executing	[4]

				secondary pyroptosis.	
	GSDME	Caspase-9	Direct Activation	GSDME pores promote cytochrome c release, activating Caspase-9.	[5,6]
	Caspase-9	Caspase-3	Direct Activation	Caspase-9 cleaves and activates Caspase-3, amplifying the apoptotic and pyroptotic cascade.	[3]

References:

[1] C.Y. Taabazuing, M.C. Okondo, D.A. Bachovchin, Pyroptosis and Apoptosis Pathways Engage in Bidirectional Crosstalk in Monocytes and Macrophages, Cell Chemical Biology 24 (2017) 507-514.e4. https://doi.org/10.1016/j.chembiol.2017.03.009.

[2] K. Tsuchiya, S. Nakajima, S. Hosojima, D. Thi Nguyen, T. Hattori, T. Manh Le, O. Hori, M.R. Mahib, Y. Yamaguchi, M. Miura, T. Kinoshita, H. Kushiyama, M. Sakurai, T. Shiroishi, T. Suda, Caspase-1 initiates apoptosis in the absence of gasdermin D, Nat Commun 10 (2019) 2091. https://doi.org/10.1038/s41467-019-09753-2.

[3] J. Yu, W. Yue, B. Wu, L. Zhang, PUMA Sensitizes Lung Cancer Cells to Chemotherapeutic Agents and Irradiation, Clin Cancer Res 12 (2006) 2928–2936. https://doi.org/10.1158/1078-0432.CCR-05-2429.

[4] Y. Wang, W. Gao, X. Shi, J. Ding, W. Liu, H. He, K. Wang, F. Shao, Chemotherapy drugs induce pyroptosis through caspase-3 cleavage of a gasdermin, Nature 547 (2017) 99–103. https://doi.org/10.1038/nature22393.

[5] C. Fu, W. Ji, Q. Cui, A. Chen, H. Weng, N. Lu, W. Yang, GSDME-mediated pyroptosis promotes anti-tumor immunity of neoadjuvant chemotherapy in breast cancer, Cancer Immunol Immunother 73 (2024) 177. https://doi.org/10.1007/s00262-024-03752-z.

[6] J. Liang, Y. He, Y. Cui, Y. Sun, G. He, Z. Zhu, X. Mao, Proteasomal inhibitors induce myeloma cell pyroptosis via the BAX/GSDME pathway, Acta Pharmacol Sin 44 (2023) 1464–1474. https://doi.org/10.1038/s41401-023-01060-3.
