# Supplementary material for: p53 Orchestrates the Immunogenic-Tolerogenic Pyroptosis Switch in Non-Small Cell Lung Cancer: A Systems Biology Approach
Source: Comput Struct Biotechnol J. 2026 Jul 21;35(1):0172. doi: 10.34133/csbj.0172 (PMC13385539; doi:10.34133/csbj.0172)
Supplement: Supplementary 1 — Tables S1 to S6 [file csbj.0172.f1.zip › Table S5.pdf]

p53 Orchestrates the Immunogenic–Tolerogenic Pyroptosis Switch in Non–Small Cell Lung Cancer: A Systems Biology Approach

Author Information

Shantanu Gupta[1,*], Daner A. Silveira[2], Rodrigo Juliani Siqueira Dalmolin[1], José Carlos M. Mombach[3], and Ronaldo F. Hashimoto[4]

Affiliations

[1] Bioinformatics Multidisciplinary Environment-BioME – Digital Metropole Institute, Federal University of Rio Grande do Norte, Natal 59076550, RN, Brazil

[2] Children's Cancer Institute, Porto Alegre, Rio Grande do Sul, Brazil

[3] Departamento de Física, Universidade Federal de Santa Maria, Santa Maria 97105-900, RS, Brazil

[4] Instituto de Matemática e Estatística, Departamento de Ciência da Computação, Universidade de São Paulo, Rua do Matão 1010, 05508-090, São Paulo - SP, Brasil

Corresponding author:

*Corresponding to: Shantanu Gupta (S.G), https://orcid.org/0000-0001-7110-6564; Email: shantanu.gupta@imd.ufrn.br ;

Table S5

Table S5: Multivariable Cox regression of pyroptosis execution-layer genes and overall survival in NSCLC (TCGA). Genes were selected based on their role in the pyroptosis-apoptosis network defined in Fig. 1. Analysis adjusted for age, sex, and tumor stage. Data processed using GEPIA3.

Gene	ENSEMBL ID	Coef	SE	HR	95% CI Lower	95% CI Upper	z	p-value
CASP3	ENSG00000164305	−0.052	0.095	0.949	0.788	1.143	−0.550	0.583
CASP9	ENSG00000132906	+0.248	0.085	1.281	1.084	1.515	+2.906	0.00366
GSDMD	ENSG00000104518	+0.022	0.068	1.022	0.895	1.167	+0.319	0.750
GSDME	ENSG00000105928	+0.085	0.043	1.089	1.002	1.184	+2.005	0.0449
NLRP3	ENSG00000162711	−0.070	0.077	0.932	0.802	1.083	−0.918	0.359

Abbreviations: Coef = regression coefficient; SE = standard error; HR = hazard ratio; CI = confidence interval.

Analysis details: Multivariable Cox regression adjusted for age, sex, tumor stage. TCGA LUAD/LUSC cohorts processed via GEPIA3.
