# Supplementary material for: p53 Orchestrates the Immunogenic-Tolerogenic Pyroptosis Switch in Non-Small Cell Lung Cancer: A Systems Biology Approach
Source: Comput Struct Biotechnol J. 2026 Jul 21;35(1):0172. doi: 10.34133/csbj.0172 (PMC13385539; doi:10.34133/csbj.0172)
Supplement: Supplementary 1 — Tables S1 to S6 [file csbj.0172.f1.zip › Table S6.pdf]

p53 Orchestrates the Immunogenic–Tolerogenic Pyroptosis Switch in Non–Small Cell Lung Cancer: A Systems Biology Approach

Author Information

Shantanu Gupta[1,*], Daner A. Silveira[2], Rodrigo Juliani Siqueira Dalmolin[1], José Carlos M. Mombach[3], and Ronaldo F. Hashimoto[4]

Affiliations

1 Bioinformatics Multidisciplinary Environment-BioME – Digital Metropole Institute, Federal University of Rio Grande do Norte, Natal 59076550, RN, Brazil

2 Children's Cancer Institute, Porto Alegre, Rio Grande do Sul, Brazil

3 Departamento de Física, Universidade Federal de Santa Maria, Santa Maria 97105-900, RS, Brazil

4 Instituto de Matemática e Estatística, Departamento de Ciência da Computação, Universidade de São Paulo, Rua do Matão 1010, 05508-090, São Paulo - SP, Brasil

Corresponding author:

*Corresponding to: Shantanu Gupta (S.G), https://orcid.org/0000-0001-7110-6564; Email: shantanu.gupta@imd.ufrn.br

Table S6

Table S6: TP53-stratified Cox regression analysis of execution-layer genes in NSCLC. Multivariable Cox proportional hazards model stratified by TP53 mutation status. HR per 1 SD increase in expression, adjusted for histology (LUAD vs LUSC). Data from TCGA PanCancer Atlas (2018).

	TP53-wildtype (n = 338)		TP53-mutant (n = 636)	
**Gene**	**HR (95% CI)**	**p-value**	**HR (95% CI)**	**p-value**
CASP3	0.89 (0.74-1.06)	>0.05	0.95 (0.83-1.09)	>0.05
CASP9	1.19 (1.06-1.34)	0.004	0.93 (0.81-1.07)	>0.05
GSDMD	1.08 (0.98-1.19)	>0.05	1.01 (0.86-1.18)	>0.05
GSDME	0.98 (0.81-1.18)	>0.05	0.98 (0.81-1.18)	>0.05
NLRP3	1.01 (0.87-1.18)	>0.05	0.87 (0.71-1.06)	>0.05

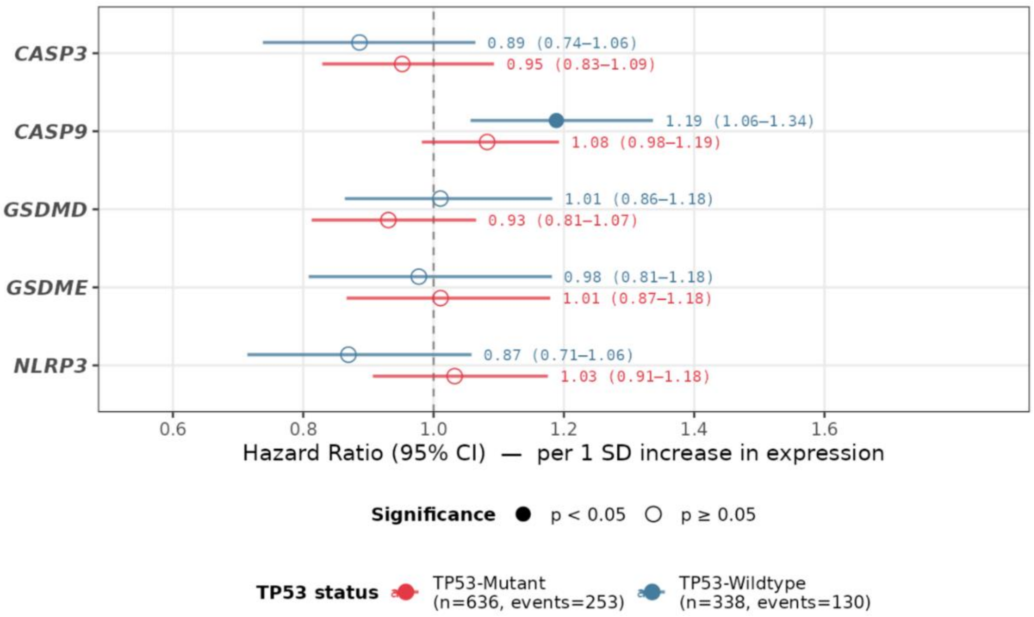

Figure S1: Forest plot of TP53-stratified survival analysis. Forest plot of multivariable Cox regression for execution-layer genes (CASP3, CASP9, GSDMD, GSDME, NLRP3) in TP53-mutant (red) and TP53-wildtype (blue) NSCLC patients. Filled circles indicate $p < 0.05$. HR per 1 SD increase in expression. TCGA PanCancer Atlas (2018).
